# The potential of circulating tumor DNA methylation analysis for the early detection and management of ovarian cancer

**DOI:** 10.1186/s13073-017-0500-7

**Published:** 2017-12-22

**Authors:** Martin Widschwendter, Michal Zikan, Benjamin Wahl, Harri Lempiäinen, Tobias Paprotka, Iona Evans, Allison Jones, Shohreh Ghazali, Daniel Reisel, Johannes Eichner, Tamas Rujan, Zhen Yang, Andrew E. Teschendorff, Andy Ryan, David Cibula, Usha Menon, Timo Wittenberger

**Affiliations:** 1 Department of Women’s Cancer, UCL Elizabeth Garrett Anderson Institute for Women’s Health, University College London, Medical School Building, Room 340, 74 Huntley Street, London, WC1E 6AU UK; 2 Gynaecologic Oncology Center, Department of Obstetrics & Gynaecology, First Faculty of Medicine & General University Hospital, Charles University, Prague, Czech Republic; 3 GATC Biotech AG, Jakob-Stadler-Platz 7, 78467 Konstanz, Germany; 4 Genedata AG, Margarethenstrasse 38, 4053 Basel, Switzerland; 5 CAS Max-Planck Partner Institute for Computational Biology, Shanghai Institute of Biological Sciences, 320 Yue Yang Road, Shanghai, 200031 China

**Keywords:** Cell-free DNA, DNA methylation, Serum DNA, Ovarian cancer, Early diagnosis, Screening, Personalized treatment

## Abstract

**Background:**

Despite a myriad of attempts in the last three decades to diagnose ovarian cancer (OC) earlier, this clinical aim still remains a significant challenge. Aberrant methylation patterns of linked CpGs analyzed in DNA fragments shed by cancers into the bloodstream (i.e. cell-free DNA) can provide highly specific signals indicating cancer presence.

**Methods:**

We analyzed 699 cancerous and non-cancerous tissues using a methylation array or reduced representation bisulfite sequencing to discover the most specific OC methylation patterns. A three-DNA-methylation-serum-marker panel was developed using targeted ultra-high coverage bisulfite sequencing in 151 women and validated in 250 women with various conditions, particularly in those associated with high CA125 levels (endometriosis and other benign pelvic masses), serial samples from 25 patients undergoing neoadjuvant chemotherapy, and a nested case control study of 172 UKCTOCS control arm participants which included serum samples up to two years before OC diagnosis.

**Results:**

The cell-free DNA amount and average fragment size in the serum samples was up to ten times higher than average published values (based on samples that were immediately processed) due to leakage of DNA from white blood cells owing to delayed time to serum separation. Despite this, the marker panel discriminated high grade serous OC patients from healthy women or patients with a benign pelvic mass with specificity/sensitivity of 90.7% (95% confidence interval [CI] = 84.3–94.8%) and 41.4% (95% CI = 24.1–60.9%), respectively. Levels of all three markers plummeted after exposure to chemotherapy and correctly identified 78% and 86% responders and non-responders (Fisher’s exact test, *p* = 0.04), respectively, which was superior to a CA125 cut-off of 35 IU/mL (20% and 75%). 57.9% (95% CI 34.0–78.9%) of women who developed OC within two years of sample collection were identified with a specificity of 88.1% (95% CI = 77.3–94.3%). Sensitivity and specificity improved further when specifically analyzing CA125 negative samples only (63.6% and 87.5%, respectively).

**Conclusions:**

Our data suggest that DNA methylation patterns in cell-free DNA have the potential to detect a proportion of OCs up to two years in advance of diagnosis and may potentially guide personalized treatment. The prospective use of novel collection vials, which stabilize blood cells and reduce background DNA contamination in serum/plasma samples, will facilitate clinical implementation of liquid biopsy analyses.

**Electronic supplementary material:**

The online version of this article (doi:10.1186/s13073-017-0500-7) contains supplementary material, which is available to authorized users.

## Background

Three-quarters of ovarian cancers (OC) are diagnosed when the tumor has spread into the abdomen and long-term survival rates of these women are low (10–30%) [1].

High-grade serous (HGS) OC accounts for 70–80% of OC deaths and the survival figures have not changed significantly over the last few decades [2]. Early diagnosis and personalized treatment still remain the biggest unmet needs in combating this devastating disease [2].

A number of OC biomarkers have been studied in the past. Among these, CA125, which was discovered more than 30 years ago [3], is still the “gold standard,” despite a modest positive predictive value when interpreted using a defined cut-off of 35 IU/mL [4], which has also been used as a reference standard in our work. Recently, the 35 most promising OC biomarkers were evaluated in the Prostate, Lung, Colorectal, and Ovarian (PLCO) Cancer Screening Trial. The markers were tested in samples taken up to six months before OC diagnosis from 118 women and 951 age-matched controls and at a fixed specificity of 95%, CA125 sensitivity out performed all 35 markers [5]. However, the performance of CA125 dropped dramatically when samples taken > 6 months before diagnosis were evaluated [5]. Recently, we demonstrated that the performance of the Risk of Ovarian Cancer Algorithm (ROCA), based on CA125 serial profile, demonstrates superior performance characteristics during screening [6, 7]. CA125 kinetics are also increasingly being explored in women undergoing neoadjuvant chemotherapy (NACT) for predicting disease response and outcome [8–11]. Both require serial blood sampling, which, in the case of differential diagnosis, is never available in patients presenting clinically.

The vast majority of protein-based tumor markers are produced not only by cancerous but also non-neoplastic normal cells; CA125 is produced by mesothelial cells (i.e. peritoneum and pleura) and hence benign or inflammatory processes can result in aberrant elevations of serum CA125.

Recently, DNA-based markers, shed from tumor cells, have shown great promise in monitoring treatment response and predicting prognosis [12–16]. However, efforts to characterize the cancer genome have shown that only a few genes are frequently mutated in most cancers and that the location of the genetic mutation site differs across individuals with specific tumor types. Hence, the detection of somatic mutations is limited to patients that harbor a predefined set of mutations. The necessity of prior knowledge regarding specific genomic composition of an individual’s tumor tissue is one of the limiting factors when using these “liquid biopsy” approaches for early detection or differential diagnosis of a pelvic mass. Current sequencing technology allows for the detection of a mutant allele fraction of 0.1% (which is one mutant molecule in a background of 1000 wild-type molecules) [12, 17].

The development of a cell-free DNA based test for the early detection of cancer poses two major challenges: (1) low abundance of cancer-DNA in the blood; and (2) high levels of “background DNA” (shed from white blood cells [WBC] [18]) in serum samples that are separated from blood cells after significant time intervals. This is an issue with most currently available population cohort biobanks which could be used for the validation of potential screening markers using samples that have been banked years in advance of diagnosis.

Alteration of DNA methylation (DNAme) is: (1) an early event in cancer development [19–22]; (2) more frequently observed than somatic mutations; and (3) centered around specific regions, i.e. CpG islands [23]. Together with its chemical and biological stability, the detection of aberrant DNAme patterns in serum or plasma provide a novel strategy for cancer diagnosis as evidenced by several proof of principle studies [24–34]. DNAme analysis allows for the detection of specific patterns (i.e. full methylation of all linked 7–16 CpGs in a region of 120–150 bp) as opposed to single point mutations (e.g. in the *TP53* gene) which is key to improving both the performance characteristics of the test and the detection limit of the assay. Plasma *SEPT9* methylation analyses—currently the only cell-free DNA assay which is available for cancer screening in the clinical setting—demonstrates a specificity of 79% and a sensitivity of 68% for detection of colon cancers [31]. The clinical potential of serum/plasma-based cell-free DNA analysis is further exemplified by maternal plasma cell-free DNA testing for fetal trisomy which has already been integrated into clinical practice and demonstrates a higher sensitivity and a lower false-positive rate compared to imaging-based techniques [35].

We have employed two different epigenome-wide approaches to identify the most promising DNAme-based markers that discriminate OC vs benign pelvic conditions, developed serum tests using the discovered markers, and validated their performance in relation to the serum OC marker CA125.

## Methods

### Patients and sample collection

**Fig. 1:**
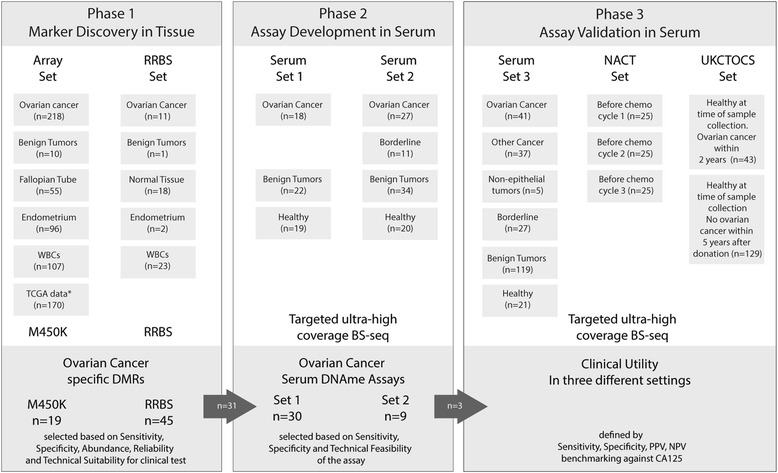
Study design. Using two different epigenome-wide technologies, 699 human tissue samples have been analyzed to identify a total of 31 regions whose methylation status has been analyzed in two serum sets consisting of 151 serum samples. Three markers have been validated in three independent settings: serum set 3, which consisted of 250 serum samples, from women with various benign and malignant conditions of the female genital tract. NACT set, consisting of serial samples from women with advanced stage ovarian cancer before and during chemotherapy. UKCTOCS (United Kingdom Collaborative Trial of Ovarian Cancer Screening) set which included serum samples from those 43 of the 101,539 women in the control arm who developed OC within 2 years; for each case, three control women who did not develop OC within 5 years of sample donation have been matched

We analyzed tissue samples from a total of 699 volunteers and 648 serum samples from a total of 598 volunteers (the 25 patients who underwent NACT provided three serial samples) in seven independent sets (Fig. 1).

#### Array set:

OC samples [36, 37], WBC samples [38], and Fallopian Tube samples [19] have previously been described. Ten benign pelvic tumors (two endometriosis-ovarian cysts, one fibroma, two papillary serous cystadenomas, one mucinous cystadenoma, two serous cystadenomas, one mucinous cystadeonoma with Brenner tumor, and one dermoid cyst), 96 endometrial samples [36] (Haukeland University Hospital, Bergen, 52 patients with primary and metastatic samples equaling 87, eight benign endometrial (all hyperplasia) and one cell line), and 170 samples (38 colon [COAD controls], 50 liver [LIHC controls], 75 lung [LUSC and LUAD controls], seven rectum [READ controls]) from the publicly available The Cancer Genome Atlas (TCGA) repository were analyzed.

#### Reduced representation bisulfite sequencing (RRBS) set:

Eleven prospectively collected invasive epithelial OC samples (HGS, *n* = 8; low grade serous, *n* = 1; endometrioid, *n* = 1; mucinous, *n* = 1; mean age = 54.7 years), one benign tumor (papillary serous cystadenoma; age = 86 years), 18 non-neoplastic tissue samples (breast, *n* = 7 and adnexal, *n* = 11; mean age = 60.2 years), two non-neoplastic endometrial tissues (mean age = 68 years), and 23 WBC samples (breast cancer patients, *n* = 10 and OC patients, *n* = 13 [11 of which match corresponding OC tissue samples, one matches corresponding normal endometrial sample, and one matches normal ovarian sample]; mean age = 57.8 years) were assessed by RRBS. All samples were collected prospectively at the University College London Hospital in London and the Charles University Hospital in Prague.

For serum sets 1–3 and the NACT serum set, women attending the University College London Hospital in London and the Charles University Hospital in Prague were invited, a written consent obtained, and 20–40 mL blood taken (VACUETTE® Z Serum Sep Clot Activator tubes, Cat. 455071, Greiner Bio One International GmbH), centrifuged at 3000 rpm for 10 min with serum stored at – 80 °C.

#### Serum set 1:

Serum samples from the following volunteers were collected (at the time of diagnosis, before treatment):

- healthy volunteers (*n* = 19, mean age = 41.1 years);
- women with benign pelvic masses (*n* = 22, mean age = 41.3 years) with the following histologies: endometriosis (*n* = 6), fibroids (*n* = 5), hydrosalpinx (*n* = 1), serous cystadenoma (*n* = 5), and mucinous cystadenoma (*n* = 5);
- patients with OCs (*n* = 18, mean age = 62.2 years): endometrioid (*n* = 2) and clear cell (*n* = 1) and HGS (*n* = 15) OCs; 10 and 8 women had a stage I/II and stage III/IV ovarian cancer, respectively.

#### Serum set 2:

Serum samples from the following volunteers were collected (at the time of diagnosis, before treatment):

- healthy volunteers (*n* = 20, mean age = 42.8 years);
- women with benign pelvic masses (*n* = 34, mean age = 40.0 years) with the following histologies: endometriosis (*n* = 7), fibroids (*n* = 8), pelvic inflammatory disease or pelvic abscess (*n* = 9), serous cystadenoma (*n* = 5), and mucinous cystadenoma (*n* = 5);
- patients with borderline ovarian tumors (*n* = 11, mean age = 47.3 years): mucinous (*n* = 6) and serous (*n* = 5) borderline tumors;
- patients with ovarian cancers (*n* = 27, mean age = 62.9 years): endometrioid (*n* = 3), clear cell (*n* = 3), mucinous (*n* = 2) and HGS (*n* = 19) OCs; 10 and 17 women had a stage I/II and stage III/IV OC, respectively.

#### Serum set 3:

Serum samples from the following volunteers were collected (at the time of diagnosis, before treatment):

- healthy volunteers (*n* = 21, mean age = 50.8 years);
- women with benign pelvic masses (*n* = 119, mean age = 41.4 years) with the following histologies: endometriosis (*n* = 21), fibroids (*n* = 21), pelvic inflammatory disease or pelvic abscess (*n* = 7), serous cystadenoma (*n* = 20), mucinous cystadenoma (*n* = 20), and dermoid cysts (*n* = 30);
- patients with borderline ovarian tumors (*n* = 27, mean age = 57.1 years): mucinous (*n* = 7) and serous (*n* = 20) borderline tumor;
- patients with non-epithelial tumors (*n* = 5, mean age = 55.8 years): granulosa cell tumors;
- patients with non-OCs (*n* = 37, mean age = 58.3 years): cervical (*n* = 10), endometrial (*n* = 20), and colorectal (*n* = 7) cancers;
- Patients with OCs (*n* = 41, mean age = 59.6 years): endometrioid (*n* = 3) and clear cell (*n* = 5), mucinous (*n* = 4) and HGS (*n* = 29) OCS; 16 and 25 women had a stage I/II and stage III/IV OC, respectively.

CA125 analysis was performed using the CA125 Cobas immunoassay and platform (Roche Diagnostics, Burgess Hill, UK) by staff who had no access to clinical or DNAme data.

#### NACT set:

Patients (*n* = 25) at the Gynaecological Oncology Centre in Prague deemed not to be suitable for upfront surgery were recruited. The average age was 62.8 years. HGS OCs were the most prevalent histology (*n* = 23) and the remaining two patients had clear cell OCs. Eighteen and seven patients presented with a stage IIIC and IV OC, respectively. Twenty-four patients received Carboplatin-Paclitaxel combination chemotherapy and one patient received Carboplatin only. All but two patients had interval debulking surgery. Among the 23 patients, 14 had no residual disease, five had macroscopic residual disease, and four had microscopic residual disease (i.e. tumor reaches the edge of at least one of the resected specimens, according to TNM classification). Twelve patients were deemed to be platinum-sensitive (no recurrence within six months after successful completion of neoadjuvant and adjuvant chemotherapy and interval debulking surgery) and eight patients were deemed to be platinum-refractory (*n* = 2, no response to chemotherapy or progression on chemotherapy) or platinum-resistant (*n* = 6, recurrence within six months after successful completion of neoadjuvant and adjuvant chemotherapy and interval debulking surgery). For five patients, no data were available on platinum-sensitivity.

All serum samples were collected prospectively at the Charles University Hospital in Prague. Each patient provided three samples at the following time-points:

- at the time of histological diagnosis, before chemotherapy;
- three weeks after the first cycle of chemotherapy (immediately before the second cycle);
- three weeks after the second cycle of chemotherapy (immediately before the third cycle).

CA125 analysis was performed using the CA125 Cobas immunoassay and platform (Roche Diagnostics, Burgess Hill, UK).

#### UKCTOCS set:

Among the 202,546 women, 101,359 women were randomized into the control arm of UKCTOCS (ClinicalTrial.gov registration, NCT00058032) between 2001 and 2005 [6, 7, 39]. Forty-three women developed an invasive epithelial OC within 2 years of serum sample donation and had at least 4 mL of non-hemolyzed serum available. Twenty-six, two, two, one, five, and seven women developed a HGS, mucinous, endometrioid, clear cell, carcinosarcoma, and a carcinoma not otherwise specified, respectively. The average age at sample donation was 63.9 years. Among the 43 women, 19 women were diagnosed within one year and 24 women were diagnosed 1–2 years after sample donation. Thirteen and 30 women were diagnosed with a stage I/II and stage III/IV cancer, respectively. For each of the 43 cases, three women who did not develop any cancer within the first five years after recruitment were matched with respect to age at recruitment, center, and month of recruitment (controls, *n* = 129) (see Additional file 1: Figure S1).

Blood samples from all UKCTOCS volunteers were spun down for serum separation after being couriered at room temperature to the central laboratory and were aliquoted and stored in liquid nitrogen vapor phase until they were thawed for this study. Only 1 mL of serum per UKCTOCS volunteer was available for cell-free DNA analysis. CA125 analysis was performed using the CA125 Cobas immunoassay and platform (Roche Diagnostics, Burgess Hill, UK). The study was approved by the local research ethics committees: UCL/UCLH Biobank for Studying Health & Disease NC09.13). All patients provided written consent for samples to be used in ethically approved secondary studies.

### Isolation and bisulfite modification of DNA

DNA was isolated from tissue and serum samples at GATC Biotech (Konstanz, Germany). Tissue DNA was quantified using NanoDrop and Qubit (both Thermo Fisher Scientific, USA); the size was assessed by agarose gel electrophoresis. Serum DNA was quantified using the Fragment Analyzer and the High Sensitivity Large Fragment Analysis Kit (AATI, USA). DNA was bisulfite converted at GATC Biotech.

### DNAme analysis in tissue

Genome-wide methylation analysis was performed either by the Illumina Infinium Human Methylation 450 K beadchip array (Illumina Inc., USA, WG-314-1003) as previously described [37, 38] or using RRBS at GATC Biotech. For the 450 K methylation data, we developed a pipeline in order to select the most promising cancer-specific differentially methylated regions (DMRs) that are most likely to fulfil the strict specificity criteria of a serum-based test (Additional file 2).

For RRBS, DNA was digested by the restriction endonuclease MspI that is specific for the CpG-containing motif CCGG; a size selection of the library provides an enhanced coverage for the CpG-rich regions including CpG islands, promoters, and enhancer elements [40, 41]. The digested DNA was adapter ligated, bisulfite-modified, and polymerase chain reaction (PCR)-amplified. The libraries were sequenced on Illumina’s HiSeq 2500 with 50 bp or 100 bp paired-end mode. Using Genedata Expressionist® for Genomic Profiling v9.1, we have established a bioinformatics pipeline for the detection of cancer-specific DMRs. The most promising DMRs have been taken forward for the development and validation of serum-based clinical assays (Additional file 2).

### Targeted ultra-high coverage bisulfite sequencing of serum DNA

Targeted bisulfite sequencing libraries were prepared at GATC Biotech. In brief, bisulfite modification was performed with 1 mL serum equivalent. Modified DNA was used to test up to three different markers using a two-step PCR approach. Ultra-high coverage sequencing was performed on Illumina’s MiSeq or HiSeq 2500 with 75 bp or 125 bp paired-end mode (Additional file 2).

### Statistical analyses

**Fig. 2:**
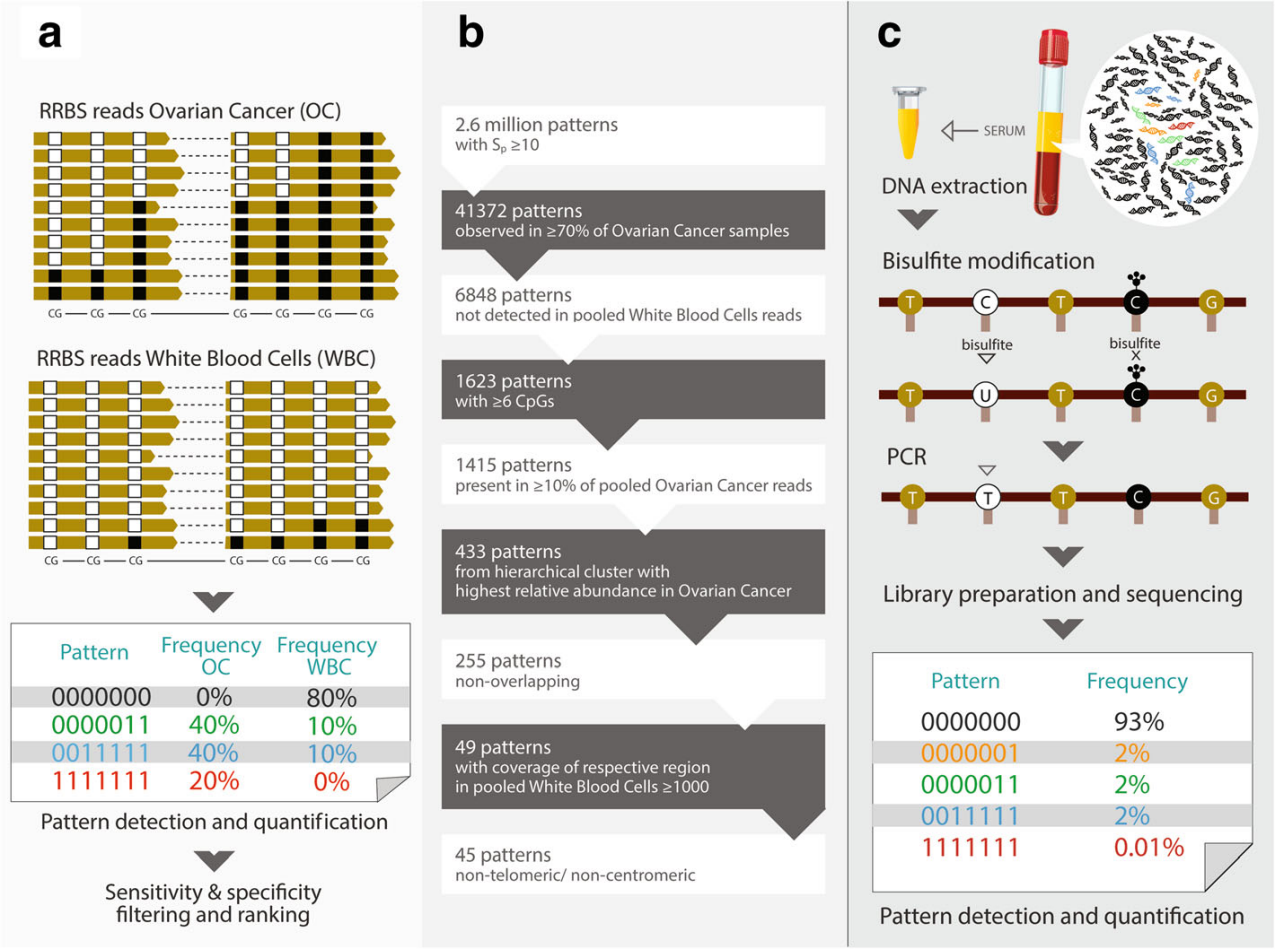
Principles of methylation pattern discovery in tissue and analyses in serum. RRBS was used in tissue samples in order to identify those CpG regions for which methylation patterns discriminate OC from other tissues, in particular blood cells which are the most abundant contaminant of cell-free DNA. An example of region #141 is provided which is a 136-bp region containing seven linked CpGs. The cancer pattern consists of reads in which all linked CpGs are methylated, indicated by “1111111” (**a**). **b** The tissue RRBS data have been processed through a bioinformatic pipeline in order to identify the most promising markers. **c** The principles of the serum DNA methylation assay are demonstrated

For DMR discovery, the data analysis pipelines are described within the respective sections in the Additional file 2. In brief, Genedata Expressionist® for Genomic Profiling was used to map reads to human genome version hg19, identify regions with tumor-specific methylation patterns, quantify the occurrence of those patterns, and calculate relative pattern frequencies per sample. Pattern frequencies were calculated as number of reads containing the pattern divided by total reads covering the pattern region. To find tumor-specific methylation patterns, we first determined the methylation pattern frequencies of all observed patterns in relevant genomic regions in different tissues. The algorithm that we developed scans the whole genome and identifies regions that contain at least ten aligned paired-end reads. These read bundles are split into smaller regions of interest which contain at least four CpGs in a stretch of, at most, 150 bp. For each region and tissue/sample, the absolute frequency (number of supporting reads) for all observed methylation patterns was determined (Fig. 2a). This led to tens of millions of patterns per tissue/sample. The patterns were filtered in a multi-step procedure to identify the methylation patterns which specifically occur in tumor samples. In order to increase sensitivity and specificity of our pattern discovery procedure, we pooled reads from different tumor or WBC samples, respectively, and scored patterns based on over-representation within tumor tissue. The results were summarized in the specificity score Sp, which reflects the cancer specificity of the patterns. After applying a cut-off of Sp ≥ 10, 2.6 million patterns for OC remained and were further filtered according to the various criteria demonstrated in Fig. 2b (and Additional file 2).

For the filtered unique cancer-specific patterns for OC identified in the Array (*n* = 19) and RRBS (*n* = 45) approach, respectively, bisulfite sequencing primers have been designed and technically validated, eventually leading to 31 candidate markers (Additional file 3: Table S1). Quantification and analysis of DNAme patterns were performed by staff who, at the time of analysis, had no access to CA125 data or clinical information. Furthermore, staff who performed the CA125 analyses had no access to clinical information at the time of the analyses. Only samples with valid values in the respective DNAme marker (no samples were excluded based on coverage) and CA125 assays (all samples had a valid value) were included in the calculation of the respective sensitivities and specificities. The 95% CI intervals for sensitivity and specificity have been calculated according to the efficient-score method [42]. Differences in pattern frequencies or coverage have been analyzed using the Mann–Whitney U test.

## Results

The samples, techniques, and purpose of the three phases—marker discovery, assay development, and test validation—are summarized in Fig. 1.

### DNA methylation marker discovery in tissue

We have used two independent epigenome-wide approaches in order to discover DMRs which have the potential to diagnose OC with high sensitivity and specificity. First, Illumina Infinium Human Methylation450 BeadChip Array (450 K) technology was used to interrogate the methylation status of ~ 485,000 genomic sites in 218 OC [36] and 438 control samples (Fig. 1 and Additional file 2). A set of 19 high scoring and ranking DMRs were selected for targeted-BS-based serum assay development. Additional file 1: Figure S2 shows an example of a selected top DMR (reaction #228). Second, based on RRBS, we developed a method for methylation quantification that takes advantage of the sequencing-specific information (i.e. the individual methylation status of all sequenced CpGs on every single DNA molecule) to overcome the challenges of using serum-based samples (i.e. relatively few tumor derived molecules in a large background of non-tumor DNA). To achieve sufficient specificity in this setting, our algorithm selects markers that are combinations of four or more CpGs on a single molecule, which show tumor specific methylation. While “background methylation” might be observed on each of these CpGs and also in WBC DNA, for example, it is much less likely that such background methylation of all measured CpGs will be observed in a single DNA molecule derived from WBCs. The analysis of single molecules also enabled us to select patterns that were not observed in any of the WBC samples analyzed, i.e. had 100% specificity in our discovery tissue sample set. Further, to achieve sufficient sensitivity in a liquid biopsy test, we restricted our markers to CpG patterns within a 150-bp window, which would allow for good PCR amplification as well as the increased likelihood of obtaining DNA fragments containing all required CpGs in apoptotic or necrotic, respectively, circulating tumor DNA (ctDNA). Finally, we generalized our algorithm so that it would also detect methylation patterns that are hypomethylated in tumors or heterogeneously methylated, respectively. This approach, together with some additional selection criteria described in Fig. 2 and Additional file 2, led to 45 marker candidates that could be utilized for the development of DNAme assays suitable for liquid biopsies testing.

Further analysis of all patterns occurring within the marker regions revealed that, while the selected, fully methylated patterns were generally more specific, truncated versions of these fully methylated patterns within the same regions (i.e. overlapping patterns including other, more, or less CpGs, respectively) showed very similar pattern frequencies in the samples analyzed (Additional file 1: Figure S3). Patterns from these regions containing one or more unmethylated CpGs were generally less specific. Heterogeneously methylated patterns in other regions were also detected (not shown), but have been filtered out in subsequent steps shown in Fig. 2.

### Serum DNAme assay establishment

We used ultra-deep BS sequencing (Fig. 2c) to develop serum assays for the candidate regions in 59 serum samples from Set 1 (Fig. 1 and Additional file 1: Figure S4 and Additional file 2). Based on sensitivity and specificity (assessed by area under the receiver operating characteristic curve [AUC]), nine markers have been selected for further validation in Set 2 (*n* = 92; Additional file 1: Figure S5). In Sets 1 and 2 combined, the specificity and sensitivity of the top four candidate markers referred to regions #141, #144, #204, and #228 (#228 was only analyzed in Set 2) to discriminate HGS OC from healthy women or those with a benign pelvic mass was 95.7%/42.4%, 93.5%/48.5%, 100%/25.0%, and 100%/36.8%, respectively (pattern frequency thresholds were set at 0.0008, 0.0001, 0.0001, and 0.0001, respectively). Interestingly, region #144 has already been defined as a promising cell-free DNA marker for cancer, particularly in OC [43, 44]. For three (i.e. #144, #204, and #228) of these four regions, CpGs were analyzed on the 450 k methylation array; using these data we demonstrated that aberrant methylation can already be detected in early stage cancers (i.e. stage I and II; Additional file 1: Figure S6). Due to limited serum volume in our validation sets, we chose a combination of three markers. The combination of regions #141, #204, and #228 (at least one of these regions with a pattern frequency above the aforementioned threshold) resulted in a 98.1% specificity and a 63.2% sensitivity. These regions are linked to genes COL23A1, C2CD4D, and WNT6, respectively.

### Clinical validation of the serum DNAme assay

**Fig. 3:**
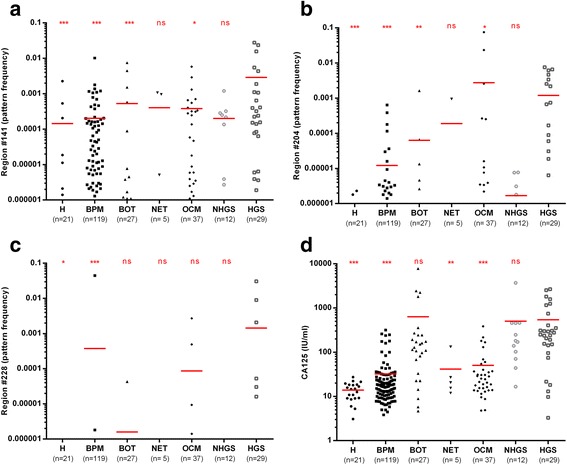
Serum DNA methylation analysis in women with benign and malignant conditions of the female genital tract. Pattern frequencies for the different regions and CA125 levels analyzed in serum set 3 samples are shown and *horizontal red bars* denote the mean (**a**–**d**; ns not significant; *p < 0.05, **p < 0.01, ***p < 0.001; Mann–Whitney U test compared to HGS; H healthy, BPM benign pelvic mass, BOT borderline tumors, NET non-epithelial tumors, OCM other cancerous malignancies, NHGS non-high grade serous ovarian cancers, HGS high grade serous, OC ovarian cancers)

We validated the combination of the three markers in Set 3 (Fig. 3a–c) alongside the CA125 serum marker (Fig. 3d). The average coverage (i.e. DNA strand reads by the sequencer for each sample and region) is > 500,000 (Additional file 1: Figure S7). Applying the above indicated cut-off thresholds for the three DNAme markers and 35 IU/mL for serum CA125 led to specificities of 90.7% and 87.1% and sensitivities of 41.4% and 82.8%, respectively (Table 1). Due to the fact that reaction #228 was only analyzed in Set 2, we combined Set 2 and Set 3 in order to redefine the thresholds. Whereas for #141 the threshold of 0.0008 remained unchanged, for #204 and #228 we further lowered the pattern frequency threshold to 0.00003 and 0.00001, respectively, leading to specificity and sensitivity of 91.8% and 58.3%, respectively (Table 1). Among these 48 HGS cancers (i.e. the most aggressive cancers), 6/11 (54.5%) stage I/II and 22/37 (59.5%) stage III/IV cancers were serum DNAme-positive. Importantly, there was no overlap between the DNAme-positive and CA125-false positive controls (Table 2).

**Table 1:** Specificities and sensitivities to discriminate high grade serous ovarian cancers from healthy and benign pelvic mass. Based on Serum Set 1&2 analyses cut-off thresholds of 0.0008, 0.0001 and 0.0001 for regions #141, #204 and #228, respectively, to discriminate HGS OC from H or BPM women were chosen and validated in Set 3. Combining Serum Sets 1-3 (note #228 was not analyzed in Set 1) the cut-off thresholds have been refined so that the final cut-offs for #141, #204 and #228 were 0.0008, 0.00003 and 0.00001 respectively; the sample was called positive if at least one of the three regions showed a pattern frequency above the cut-off. 95% CI, 95% Confidence Interval; DNAme, DNA methylation

		Specificity	Sensitivity
Set 3	CA125 (cut-off 35 IU/mL)	122/140 (87.1%; 95% CI = 80.1–92.0%)	24/29 (82.8%; 95% CI = 63.5–93.5%)
	3 DNAme-Marker Panel (thresholds based on Sets 1 and 2)	127/140 (90.7%; 95% CI = 84.3–94.8%)	12/29 (41.4%; 95% CI = 24.1–60.9%)
Set 2 & 3	3 DNAme-Marker Panel (new thresholds based on Sets 1, 2, and 3)	178/194 (91.8%, 95% CI = 86.7–95.1%)	28/48 (58.3%; 95% CI = 43.2–72.1%)

**Table 2:** The overlap between CA125-positive samples (cut-off > 35 IU/mL) and the three DNAme marker panel (using refined new thresholds, see Table 1) in HGS cancer cases and healthy (H)/benign pelvic mass (BPM) controls in serum set 3

		H and BPM		HGS	
		CA125-negative	CA125-positive	CA125-negative	CA125-positive
3 DNAme marker panel (new thresholds)	Negative	108	18	4	9
	Positive	14	0	1	15

### Serum DNAme to predict response to platinum-based NACT

**Fig. 4:**
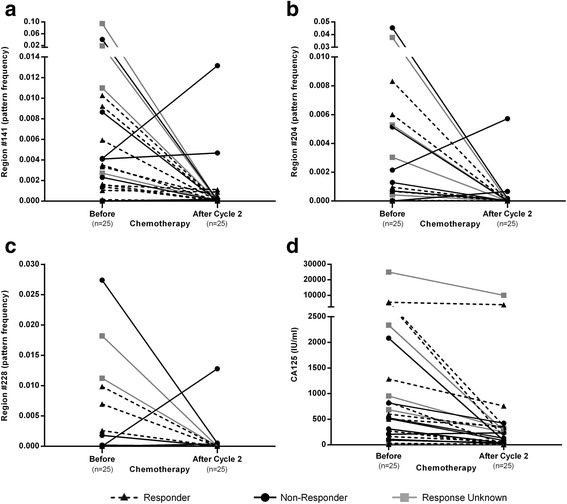
The dynamics of serum DNAme markers and CA125 as a function of exposure to Carboplatin-based chemotherapy. The changes in pattern frequency of the three markers as well as CA125 is shown before being compared after two cycles of chemotherapy (**a**–**d**) in the NACT set. Responder: no recurrence within six months after successful completion of NACT and adjuvant chemotherapy and interval debulking surgery; Non-Responder: either no response to chemotherapy or progression on chemotherapy or recurrence within six months after successful completion of NACT and adjuvant chemotherapy and interval debulking surgery

In order to further assess the cancer specificity and the dynamics of our three candidate markers in individual patients, we recruited 25 OC patients who received carboplatin-based NACT. Compared with the pre-treatment sample, all three DNAme markers decreased substantially and to a larger extent compared to CA125 after one and two cycles (Fig. 4a–d and Additional file 1: Figures S8–S10). Whereas CA125 dynamics were not a strong discriminator between chemotherapy-responders and non-responders (Table 3), serum DNAme dynamics (i.e. serum DNAme as defined in Sets 2 and 3, before chemotherapy compared to after two cycles) correctly identified 78% and 86% of responders and non-responders (Fisher’s exact test, *p* = 0.04) overall and 78% and 100% of responders and non-responders among those women who were left without residual disease after interval debulking surgery (Fisher’s exact test, *p* = 0.007) (Table 3).

**Table 3:** The changes of markers during NACT (NACT set) and whether this can predict response to chemotherapy in all patients and in those who had no macroscopic residual disease after interval-debulking surgery (R0/1)

Prediction chemosensitivity		Responder		Non-responder	
		All	R0/1	All	R0/1
CA125	Positive → negative	2/10 (20%)	2/8 (25%)		
	Positive → positive			6/8 (75%)	4/6 (66.7%)
DNAme	DNAme Positive → negative	7/9 (77.8%)	7/9 (77.8%)		
	DNAme Positive or negative → positive			6/7 (85.7%)	6/6 (100%)

CA125 concentration of < 35IU/mL was deemed negative. Definitions of DNA methylation positivity are provided in Table 1. Note that amongst the 20 patients who had chemo-sensitivity data available, they were only included in the analysis if the pre- and/or post-treatment (after cycle 2) sample were positive (i.e. in 2 and 4 patients for CA125 and DNA methylation markers, respectively, neither sample was positive and hence response or lack of response could not be assessed)

### Serum DNAme for early diagnosis of OC

**Fig. 5:**
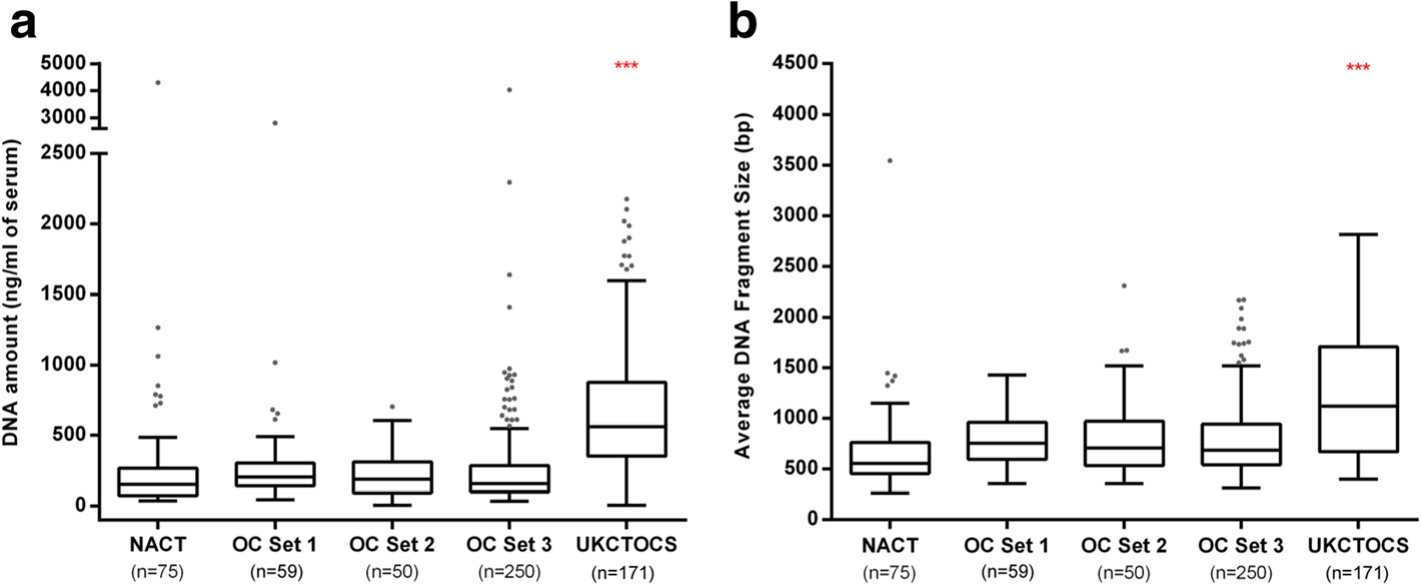
Performance of the serum DNAme marker panel in a population-based cohort for early OC diagnosis. Compared to the prospectively collected samples within the EpiFemCare Programme, UKCTOCS samples contained a significantly higher DNA concentration (**a**) and larger average DNA fragment size (**b**). As a result of this, we had to lower the cut-off for the three markers by a factor three (i.e. pattern frequency cut-off for #141, #204, and #228 is 0.00027, 0.00001, and 0.0000033, respectively). For OC Set 2, we only display the result of the 50 samples for which we have analyzed both DNA amount and fragment size (for 42 samples, we only analyzed DNA amount). In addition, in one UKCTOCS sample, the fragment size analysis failed. ***p < 0.001

In order to judge whether our marker panel is, in fact, capable of diagnosing OC early, samples predating OC diagnosis by up to two years (cases) and matched controls were used from the control (no screening) arm of the UKCTOCS cohort. The median time from venepuncture to serum separation was 21.97 h (interquartile range [IQR] = 19.91–24.34 h) for this sample set. As expected, both the amount of DNA/mL serum as well as the average DNA fragment size were substantially higher in UKCTOCS samples compared with the other samples used in this study (Fig. 5a and b). This is potentially due to DNA from WBCs leaking into the serum during the sample transport time, particularly during the warmer months of the year (Additional file 1: Figure S11). Nevertheless, a small proportion (on average, 19.9%) of the DNA consisted of smaller (50–250 bp) fragments representing DNA from apoptotic cells (including DNA from cancer cells) (Additional file 1: Figure S12). The “contaminating” majority of high-quality DNA not only dilutes the cancer signal but also skews the target sequence amplification towards WBC DNA. In order to adjust for these factors, we made an a priori decision to reduce the threshold for the three regions by a factor of 3 and split the analyses in samples above (high) and below (low) the median amount of DNA (Table 4). The three DNAme-marker panel was able to identify cases with a specificity of 88% and a sensitivity of 58%, when specifically assessed in samples with a DNA concentration lower than the median ng/uL value, and importantly which predated cancer diagnosis by up to two years (Table 4B and Additional file 3: Table S2). The sensitivity of the panel improved from 58% to 64% when exclusively assessing CA125-negative (<35 IU/mL) samples. As previously observed in the Set 3 analysis there is no overlap between CA125 and DNAme false-positive controls (Table 5). When directly comparing the performance of CA125 (applying a cut-off of 35 IU/mL) with the DNAme panel specifically in the “low” DNA samples, the DNAme panel had higher sensitivity (57.9% vs 42.1%) but lower specificity (88.1% vs 95.5%) compared to that of CA125 for the early detection of OC (Additional file 3: Table S3).

**Table 4:** Specificity and sensitivity to detect OC in pre-diagnostic UKCTOCS samples are shown for the complete sample set (A), as well as for samples split according to DNA concentration below (low DNA) (B) and above (high DNA) (C) the median and with a CA125 concentration < 35 IU/mL (CA125-negative samples) in the three groups. Definitions of DNA methylation positivity are provided in Table 1

(A)	**All samples**			**CA125-negative samples**		
	**0–2 years**	**0–1 years**	**1–2 years**	**0–2 years**	**0–1 years**	**1–2 years**
Specificity (CI%)	96.9% (90.8–98.6)	96.9% (90.8–98.6)	96.9% (90.8–98.6)	96.8% (91.5–99)	96.8% (91.5–99)	96.8% (91.5–99)
Sensitivity (CI%)	23.3% (12.3–39)	31.6% (13.6–56.5)	16.7% (5.5–38.2)	15.4% (5–35.7)	22.2% (3.9–59.8)	11.8% (2.1–37.8)
(B)	**Low DNA samples**			**CA125-negative samples**		
	**0–2 years**	**0–1 years**	**1–2 years**	**0–2 years**	**0–1 years**	**1–2 years**
Specificity (CI%)	88.1% (77.3–94.3)	88.1% (77.3–94.3)	88.1% (77.3–94.3)	87.5% (76.3–94.1)	87.5% (76.3–94.1)	87.5% (76.3–94.1)
Sensitivity (CI%)	57.9% (34.0–78.9)	55.6% (22.7–84.7)	60.0% (27.4–86.3)	63.6% (31.6–87.6)	66.7% (24.1–94.0)	60% (17.0–92.7)
(C)	**High DNA samples**			**CA125-negative samples**		
	**0–2 years**	**0–1 years**	**1–2 years**	**0–2 years**	**0–1 years**	**1–2 years**
Specificity (CI%)	90.3% (79.5–96)	90.3% (79.5–96)	90.3% (79.5–96)	90.2% (79.2–95.9)	90.2% (79.2–95.9)	90.2% (79.2–95.9)
Sensitivity (CI%)	12.5% (3.3–33.5)	30% (8.1–64.6)	0% (0–26.8)	0% (0–25.3)	0% (0–69)	0% (0–30.1)

**Table 5:** The overlap between CA125-positive samples (cut-off > 35 IU/mL) and the three DNAme marker panel (defined in Table 1) in cases and controls in the UKCTOCS “low DNA” nested case/control setting

		Controls (CA125)		Cases (CA125)	
		Negative	Positive	Negative	Positive
3 DNAme marker panel	Negative	56	3	4	4
	Positive	8	0	7	4

## Discussion

We identified cancer-specific DNAme patterns in tissue and developed serum assays which we validated in various settings. Our findings suggested that cell-free DNA has the potential to detect ovarian cancer up to two years in advance of clinical diagnosis. Nevertheless, a cell-free DNA based test will have to overcome several hurdles before clinical implementation.

In healthy individuals, cell-free DNA is present at concentrations in the range of 0–100 ng/mL and an average of 30 ng/mL [45]. DNA derived from tumor cells is shorter than that from non-malignant cells in the plasma of cancer patients [46]. Our overarching goal was to develop DNAme-based markers for early OC detection. In order to realize this aim, samples must be derived from large population-based screening studies, such as the UKCTOCS trial, that have samples banked years ahead of diagnosis. Serum samples from ~ 100,000 women need to be collected to accrue sufficient OC numbers (i.e. a range of 40–50). Within the UKCTOCS, which involved collection and banking of serum samples from over 200,000 women, whole blood samples were couriered to the central laboratory within 2–48 h. Prospectively collected blood samples were spun down 2–12 hours after collection to mimic the UKCTOCS setting. The UKCTOCS, and to a lesser extent the other prospectively collected sets, contained higher than average amounts of cell-free DNA and fragments were longer on average. Both factors reflect the leakage of WBC DNA into serum. In order to compensate for this, we aimed for an extremely high coverage but noted that four of the UKCTOCS samples had at least one of the three markers with a coverage 100,000; we had made an upfront decision not to exclude any of the samples based on lower coverage but note that this is one of the limitations of this study. Despite these complicating factors the three-DNAme marker panel outperformed CA125 using a 35 IU/mL cut-off in detecting OC early in the group of women who had a DNA concentration lower than the median ng/uL value.

In order to provide further functional proof that the newly developed serum DNAme marker panel is cancer-specific and able to indicate the presence of active OC, despite competing with high levels of background WBC DNA inherent within the trial samples analyzed, we demonstrated that our serum DNAme-dynamics correctly identified 7/9 and 6/7 Carboplatin responders and non-responders, respectively.

As we did not observe an overlap between false-positive CA125 and false-positive DNAme samples, it is probable that DNAme false positivity is largely triggered by technical artefacts as a result of extremely low thresholds down to a pattern frequency of 0.000003 (i.e. three cancer patterns in the background of 1,000,000 DNA fragments with a non-cancer pattern). Of note is that for the serum sets, which have been prospectively collected within EpiFemCare, there was a substantial age difference between women who presented with benign pelvic masses and women who presented with OC. This age skew was completely intentional as our main purpose was to benchmark DNAme markers against CA125 false-positive controls and to assess whether CA125 false-positive controls are also DNAme-false positive. The main sources of false positivity are endometriosis, pelvic inflammatory disease, and fibroids—all conditions which are substantially more prevalent (or occur exclusively) in premenopausal (i.e. younger women), whereas OC is far more prevalent in older women. False CA125-positivity can usually be explained by a CA125-producing benign condition [47].

At the UKCTOCS prevalence screen [39], the ROCA identified elevated/intermediate risk in 0.93% of women, of whom 0.9% (42/4642) were diagnosed after repeat CA125 testing, ultrasound, additional imaging, and clinical assessment. Applying the three-marker DNAme test, with a conservative (i.e. excessive background DNA will not be an issue in prospective samples) estimate of specificity and sensitivity of 90% and 60%, respectively, as a second line test to ROCA-elevated women at risk could substantially decrease the time to diagnosis in at least half the women with OC.

OC is a low prevalence disease (i.e. lifetime risk in the general female population is 1–2 per 100 women [48]). The consequence of a positive screening test is an operation under general anesthesia (i.e. laparoscopic or open) to remove one or both ovaries/Fallopian Tubes. Hence, a high specificity of the screening test is of the utmost importance because the positive predictive value strongly depends on the prevalence of the disease and the specificity of the test [34]. Using a highly sensitive marker panel, able to detect > 80% of stage I/II OCS (i.e. a combination of CA125 and HE4 [49]), to pre-screen the entire population in order to narrow down the group of women who have a high likelihood of OC followed by the cell-free DNA test is a highly promising strategy to achieve a stage shift with at least 50% of cancers (instead of 25% in the absence of screening) diagnosed in stage I/II.

In addition to the use of serum and high levels of contaminating normal DNA from blood cells, the current work has some further limitations. First, the number of samples (specifically when considering only the low-DNA samples) in the UKCTOCS cohort was limited. Second, we were unable to assess whether the panel is specific for OC or whether it may additionally detect other cancer types. In serum set 3, we also analyzed serum samples from 37 patients with non-OCs (ten, 20, and seven with cervical, endometrial, and colorectal cancers, respectively). Two of ten (20%), 5/20 (25%), and 1/7 (14%) were deemed positive based on the final three-marker panels. This may indicate that our panel also detects other cancers arising from the Mullerian tract (i.e. cervical and endometrial cancers). In order to further elaborate on the aforementioned, we assessed the TCGA data. Whereas there was no CpG site on the 450 k methylation array for region #141, for regions #204 and #228 there were two (cg15015892 and cg05021743) and one (cg22344703) CpGs, respectively, represented on the Illumina array. Also, other cancers could potentially be identified using these markers (Additional file 1: Figure S13). Third, we did not directly compare the methylation levels in the primary tumor and the matched serum samples. As we have shown (Additional file 1: Figure S6), methylation levels in the primary cancers are relatively homogenous across different stages of OCs. Hence, any differences in methylation levels detected in the serum reflect conditions such as cancer cell turnover, release of cell-free DNA via lymph-vessels into the bloodstream, and half-life in the circulation—all factors which cannot be assessed by directly measuring DNAme in the cancer.

Our method of defining tumor-specific methylation patterns and quantifying the molecules exhibiting such patterns, instead of determining methylation levels, shows promising results regarding its applicability in liquid biopsy testing. While, in this study, the most promising tumor markers were all fully methylated, the method, per se, is not biased towards hyper- or uniformly methylated patterns and, as such, is also applicable to situations where the markers of interest show hypo- or heterogenous methylation, respectively.

Overall, our study provides a proof of principle that serum DNAme markers have the potential to detect OC within two years in advance of diagnosis and may therefore be able to guide personalized OC treatment. The recent advance of purpose-made blood collection tubes that stabilize cell-free DNA and prevent leakage of DNA from blood cells [50] will facilitate clinical implementation of DNAme pattern detection in cell-free DNA as a clinical tool in cancer medicine. In addition, recent evidence demonstrates that using DNAme patterns will allow for tissue-of-origin mapping in circulating cell-free DNA [51, 52] which supports the view that a DNAme marker panel is likely to cover a number of tumor-entities.

## Conclusions

Overall, and for the first time, our study suggests that serum DNAme markers have the potential to diagnose OCs up to two years in advance of current diagnosis and may potentially enable individualized OC treatment. The recent advance of purposed blood tubes will facilitate clinical implementation of DNAme pattern detection of cell-free DNA as a clinical tool in cancer medicine.

## Additional files

Additional file 1: Figure S1. Design of the nested case-control study based on the UKCTOCS Cohort. **Figure S2.** DMR discovery with Illumina 450 K methylation arrays. **Figure S3.** Pattern counts for informative regions. **Figure S4.** Pattern frequencies for the different regions analyzed in serum set 1 samples. **Figure S5.** Pattern frequencies for the different regions analyzed in serum set 2 samples. **Figure S6.** DNA methylation for regions #144, #204, and #228 according to OC stages. **Figure S7.** Coverage (number of reads) for the three different regions analyzed in serum set 3 samples. **Figure S8.** CA125 levels measured in NACT serum set samples. **Figure S9.** Pattern frequencies for the top three reactions measured in NACT serum set samples. **Figure S10.** Coverage (number of reads) for the top three reactions measured in NACT serum set samples. **Figure S11.** Average DNA amount extracted correlates with average UK temperature. **Figure S12** The fraction (%) of small fragment (50–250 bp) DNA in the serum DNA preparation for 171 UKCTOCS samples analyzed in the study. **Figure S13.** Box plots comparing the average beta values for 450 k array probes within regions #204 and #228 between each normal (N), cancer (C) group, and white blood cell (WBC) data for OC and other 19 TCGA cancer types. (DOCX 3024 kb)

Additional file 2: Supplementary Material and Methods: Additional details of samples sets, methods, and analyses. (DOCX 75 kb)

Additional file 3: Table S1. Coordinates and primers to amplify the identified target region. **Table S2.** Performance of the serum DNA methylation marker panel in a population-based cohort to diagnose OC early. **Table S3.** Direct comparison of the performance of the CA125 and the three-marker DNAme panel to predict OC development in the UKCTOCS cohort. (DOCX 29 kb)

## Abbreviations

AUC: Area under the curve
bp: Base pairs
BPM: Benign pelvic mass
DNAme: DNA methylation
NACT: Neoadjuvant chemotherapy
H: Healthy
HGS: High grade serous
OC: Ovarian cancer
ROC: Receiver operating characteristic
WBC: White blood cell
