# Supplementary material for: The potential of circulating tumor DNA methylation analysis for the early detection and management of ovarian cancer
Source: Genome Med. 2017 Dec 22;9:116. doi: 10.1186/s13073-017-0500-7 (PMC5740748; doi:10.1186/s13073-017-0500-7)
Supplement: Supplementary file 1 — Design of the nested case-control study based on the UKCTOCS Cohort. Figure S2. DMR discovery with Illumina 450 K methylation arrays. Figure S3. Pattern counts for informative regions. Figure S4. Pattern frequencies for the different regions analyzed in serum set 1 samples. Figure S5. Pattern frequencies for the different regions analyzed in serum set 2 samples. Figure S6. DNA methylation for regions #144, #204, and #228 according to OC stages. Figure S7. Coverage (number of reads) for the three different regions analyzed in serum set 3 samples. Figure S8. CA125 levels measured in NACT serum set samples. Figure S9. Pattern frequencies for the top three reactions measured in NACT serum set samples. Figure S10. Coverage (number of reads) for the top three reactions measured in NACT serum set samples. Figure S11. Average DNA amount extracted correlates with average UK temperature. Figure S12 The fraction (%) of small fragment (50–250 bp) DNA in the serum DNA preparation for 171 UKCTOCS samples analyzed in the study. Figure S13. Box plots comparing the average beta values for 450 k array probes within regions #204 and #228 between each normal (N), cancer (C) group, and white blood cell (WBC) data for OC and other 19 TCGA cancer types. (DOCX 3024 kb) [file 13073_2017_500_MOESM1_ESM.docx]

**Supporting information for:**

**The potential of circulating tumor DNA methylation analysis for the early detection and management of ovarian cancer**

**Supplementary Figures**


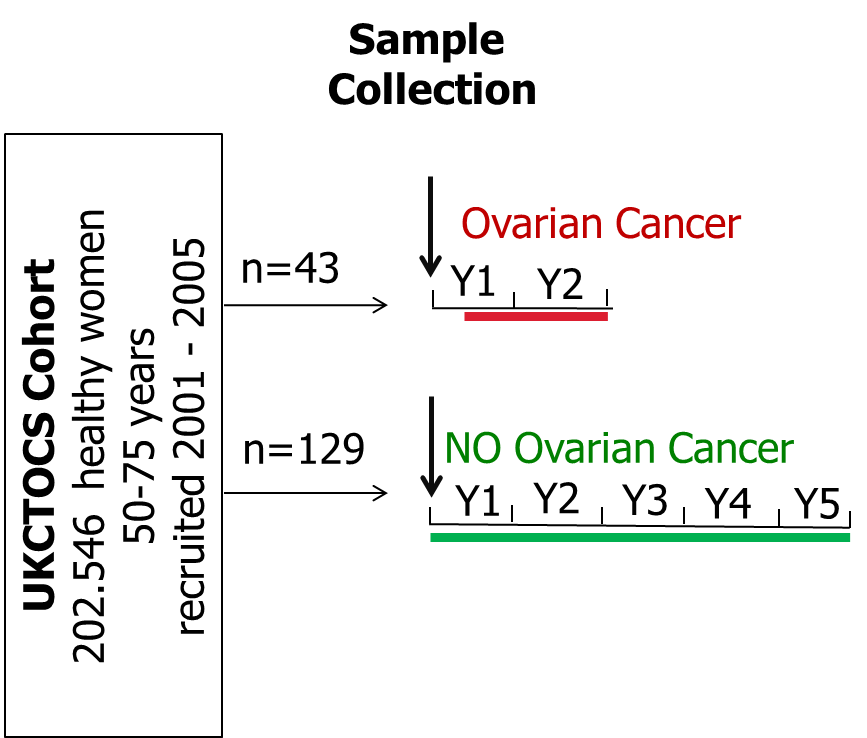


**Fig S1. Design of the nested case control study based on the UKCTOCS Cohort.** Among the total of 202,546 women, 101,359 were randomized into the control arm of UKCTOCS (ClinicalTrial.gov registration ID is NCT00058032) between 2001 and 2005, 43 of which developed an invasive epithelial ovarian cancer within 2 years of serum sample donation. For each of the 43 cases, three women who did not develop cancer within the first five years after recruitment were matched with respect to age at recruitment, center and month of recruitment (controls, n=129).

**A**


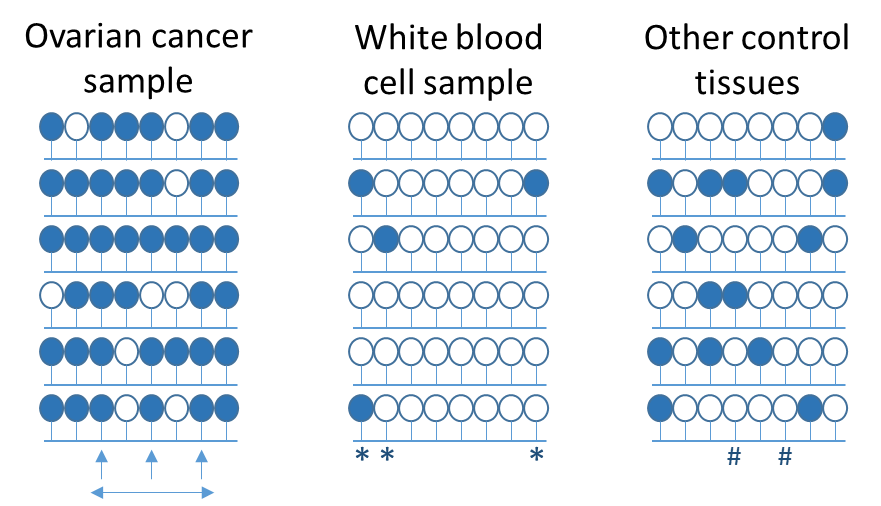


**B**


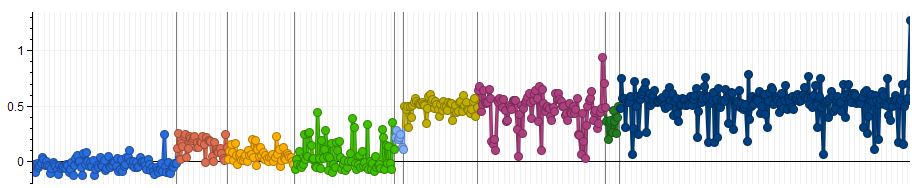

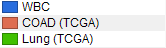

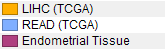

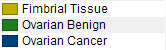


**Fig S2. DMR discovery with Illumina 450K methylation arrays.** (**A**) Schematic illustration of DMRs that are discovered by the single CpG and range approaches. Each horizontal line of lollipops indicates neighboring CpGs in a single DNA molecule extracted from the indicated tissue. Filled (blue) lollipop indicates a methylated CpG, and an unfilled (white) lollipop indicates an unmethylated CpG. 450K methylation arrays measure the ratio (% of methylation) of methylated and unmethylated molecules at a given single CpG location. See Supplementary Methods for details on the DMR discovery methods. (**B**) Example of the methylation data for a high scoring DMR. The #228 targeted BS reaction was designed for this DMR.

↑ Single CpG DMRs (high scoring); ↔Range DMR (high scoring); *Not identified as DMRs because of the methylation in WBCs, #Not identified as high scoring DMRs with single CpG approach because the methylation difference between OC and other control tissues (=colon, lung, liver, rectum, endometrium, fimbriae and benign ovarian tissue) is not large enough.


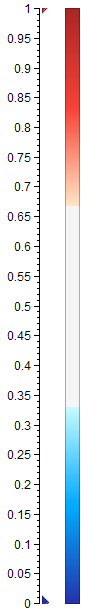


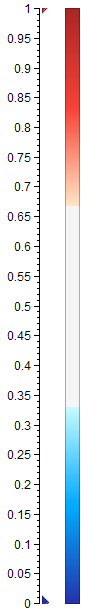

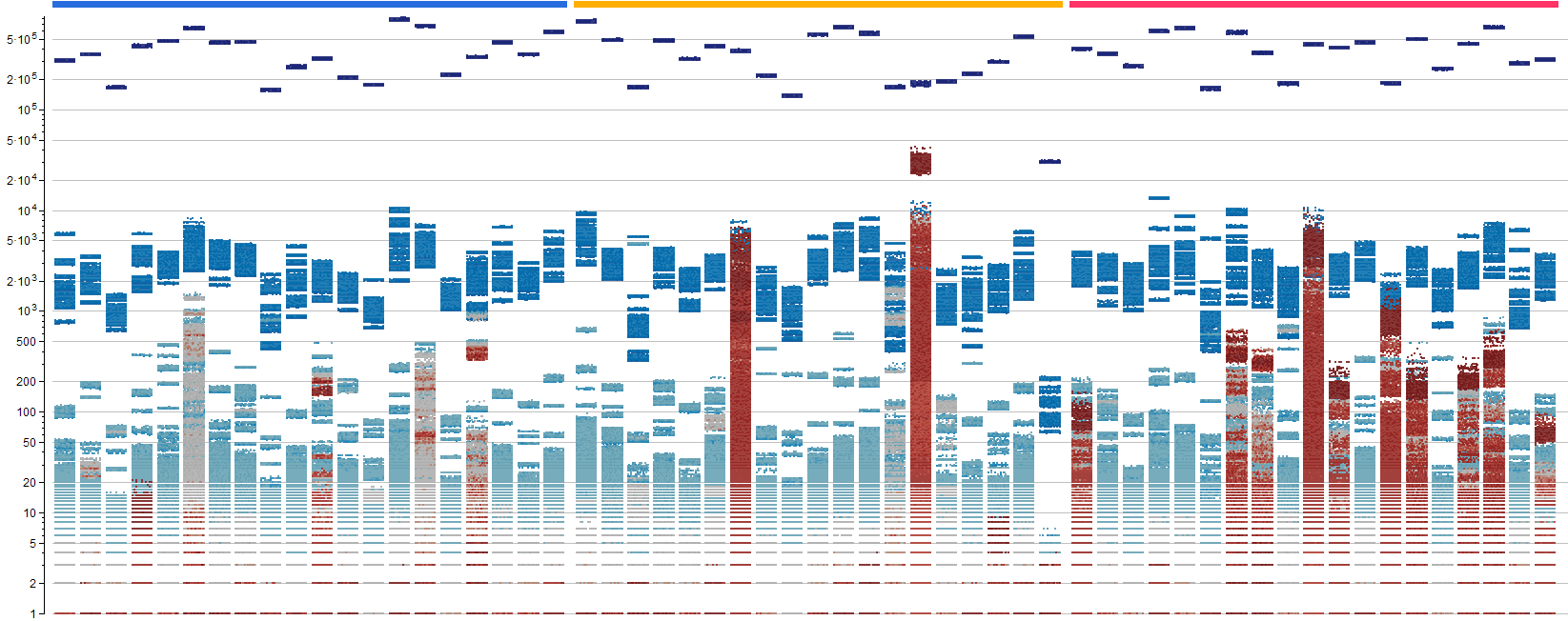


**
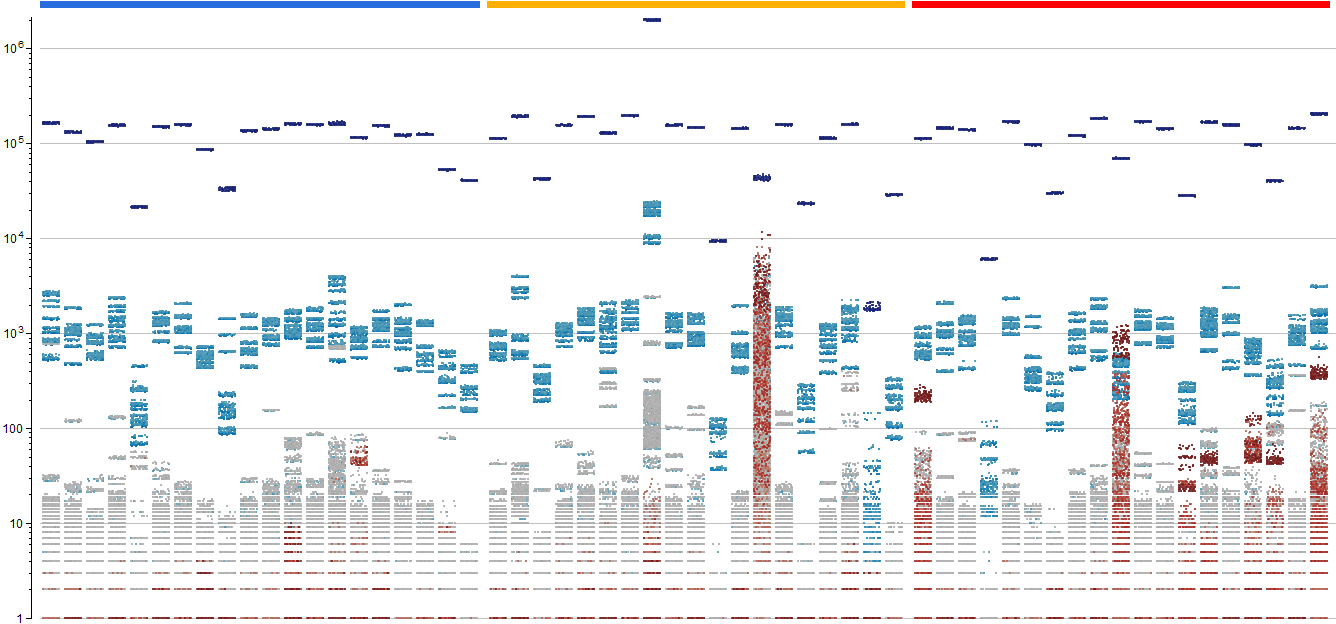
**

**
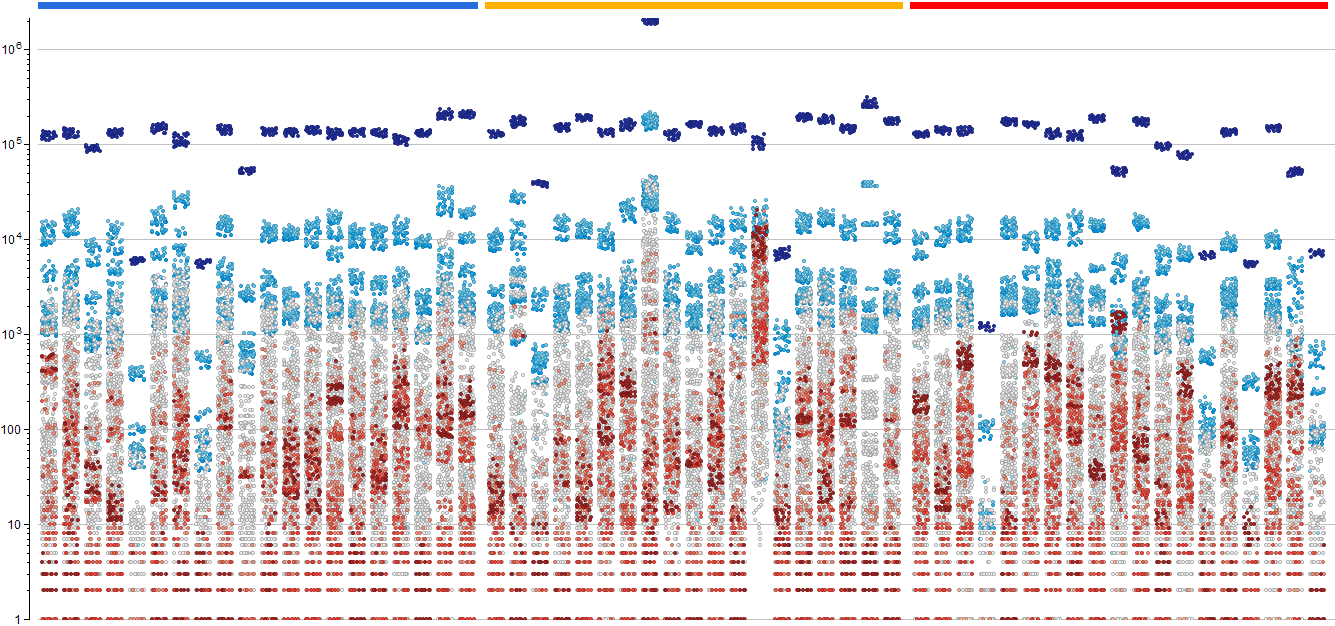
**

**Fig S3a. Absolute pattern counts for all patterns detected in the region of marker #204 (top), #228 (middle), and #141 (bottom), respectively, in Set 2 samples.** Shown are dot plots with individual dots for all of the patterns for each of the samples. The y-axis gives counts per pattern (log scale); samples are grouped into healthy (blue), ovarian cancer (orange), and high-grade serous ovarian cancer (red). Patterns are colored according to methylation level (color scale ranging from 0 to 1, with 1 = all CpGs of the pattern methylated). Patterns with similar methylation levels cluster together, for example fully methylated patterns have similar read counts, independently of the number of CpGs contained in a pattern (between 7 and 11 in this case) and in which the available CpGs are included. Fully unmethylated patterns are detected most frequently in all samples (>10^5^ counts), while patterns with a single methylated CpG are observed about 100-fold less often, largely independent of the position of the methylated CpG. Dots representing highly methylated patterns are displayed in the foreground, while unmethylated patterns are in the background for visibility reasons (blue and white dots are approximately four times more abundant than red dots).

**
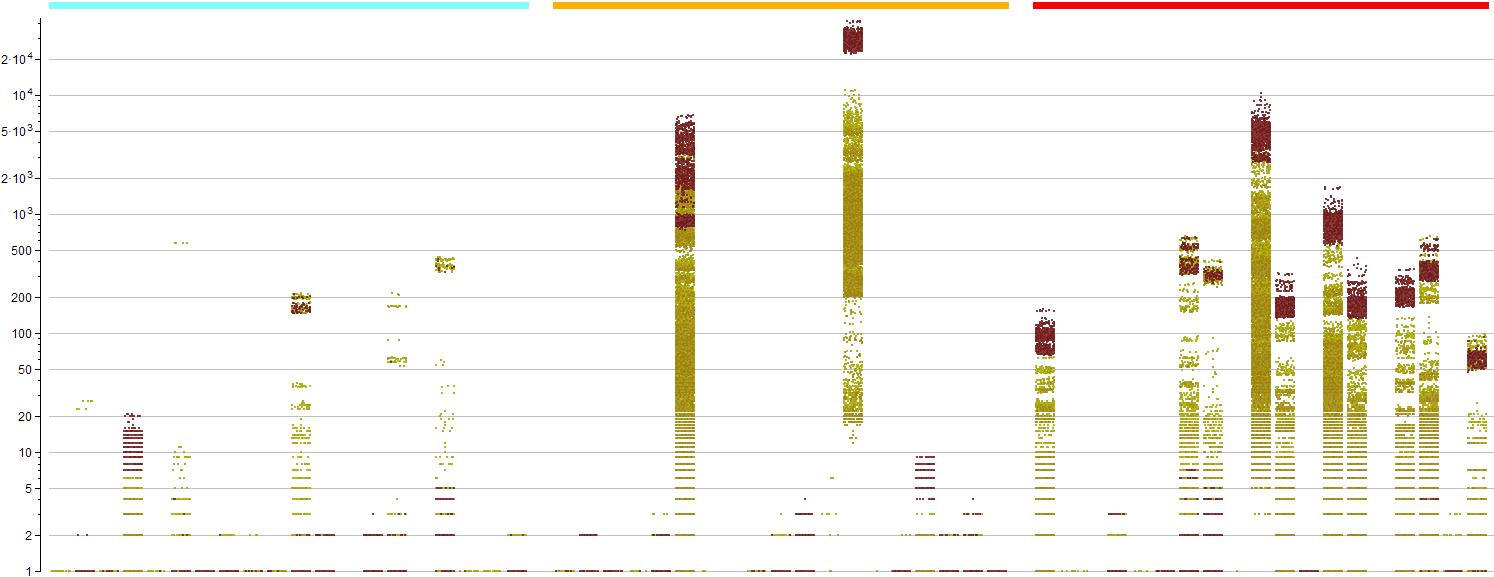
**

**
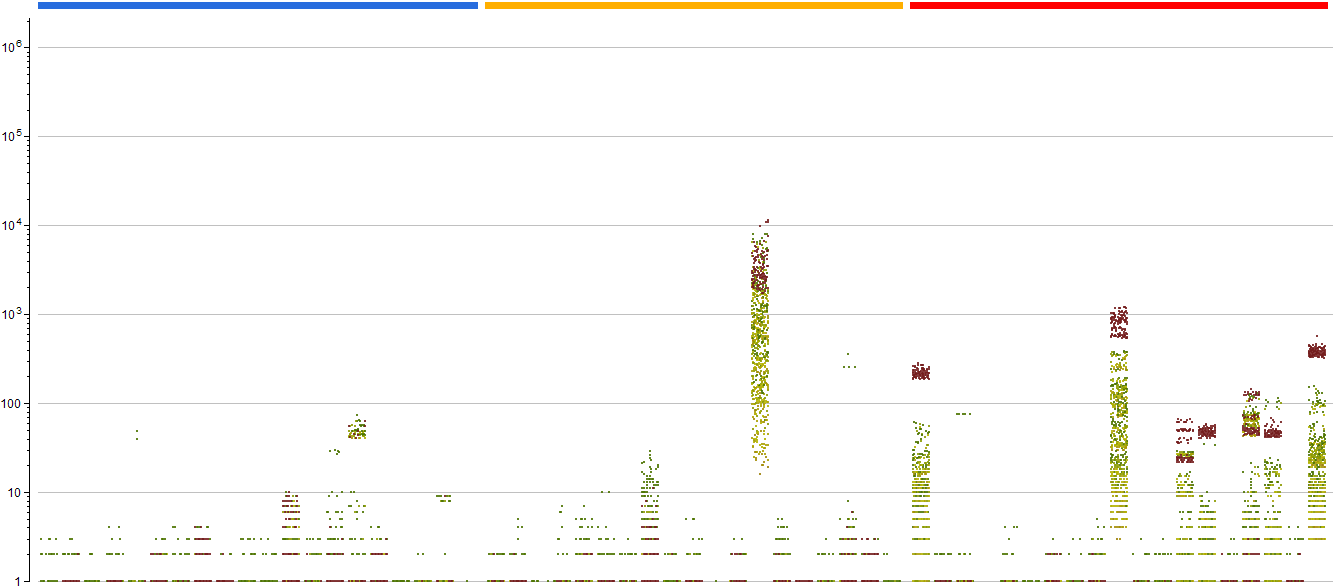
**

**
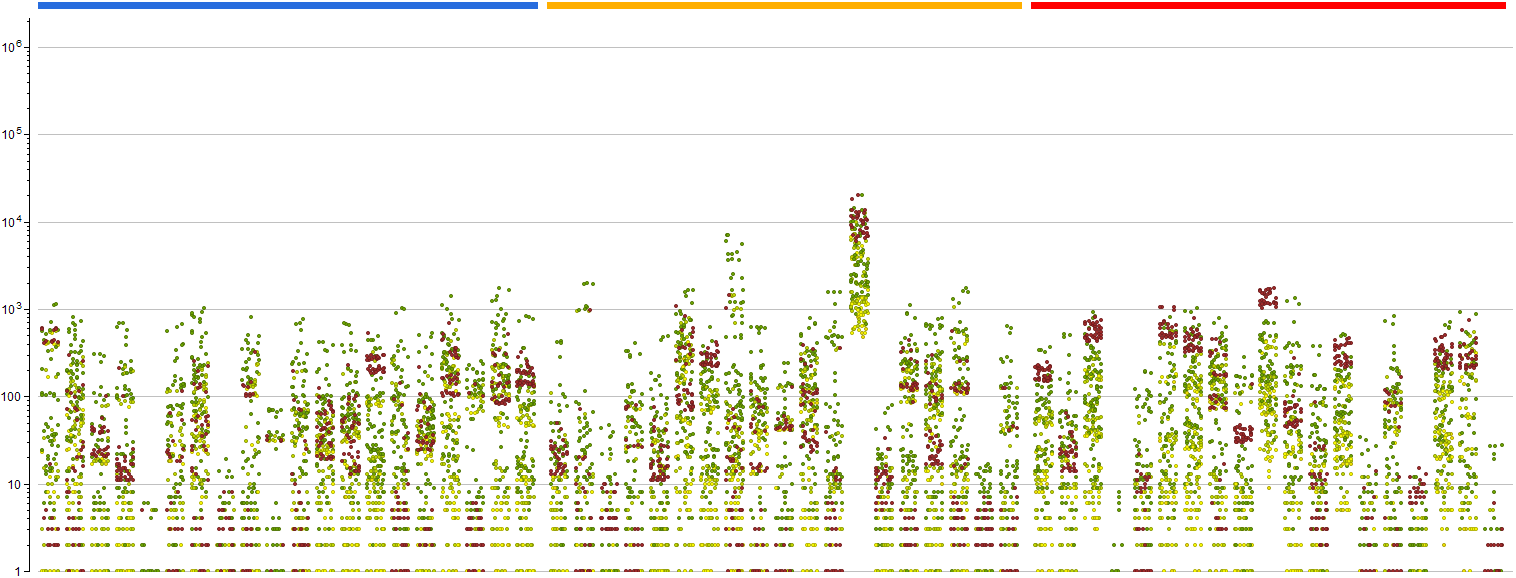
**

**Fig S3b.** Similar to Figure S3a, but showing only fully methylated patterns (red) and patterns with one CpG being unmethylated (green). In samples where highly methylated patterns are observed, fully methylated patterns are counted more frequently than patterns containing one unmethylated CpG. Differences in shading of green are due to the fact that the proportion of methylated CpGs, given a single unmethylated CpG, depends on the total number of CpGs contained within a given pattern (e.g. 0111 = 75%; 01111 = 80% methylated).


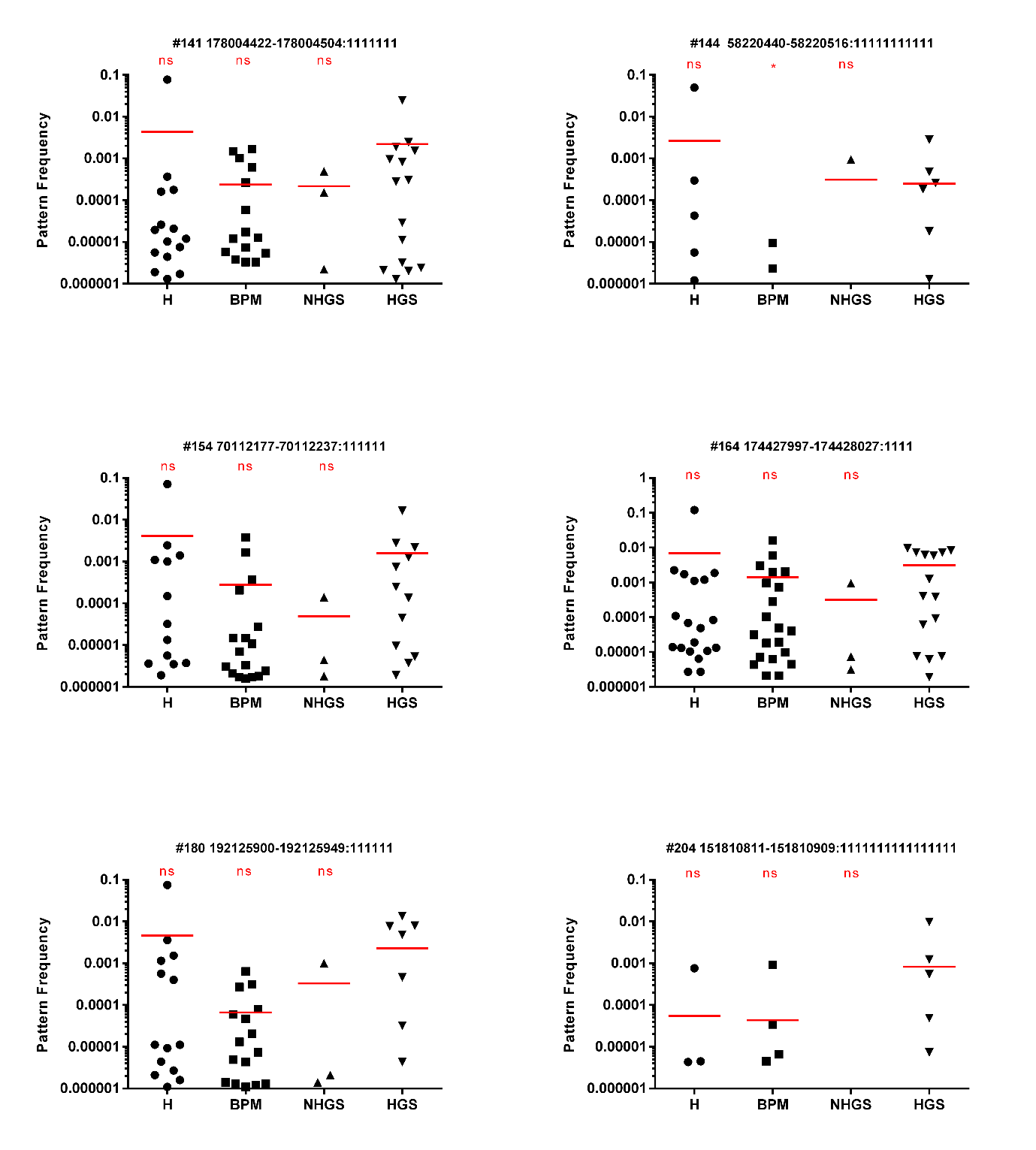


**Fig S4. Pattern frequencies for the different regions analyzed in Serum Set 1 samples.** H, Healthy; BPM, benign pelvic mass; NHGS, non-high grade serous ovarian cancers; HGS, high grade serous ovarian cancers. Horizontal red bar denotes mean. ns not significant; *p<0.05, Mann-Whitney U test compared to HGS.





**Fig S5. Pattern frequencies for the different regions analyzed in Serum Set 2 samples.** H, Healthy; BPM, benign pelvic mass; BOT, borderline tumor; NET, non-epithelial tumors, OCM, other cancerous malignancies; NHGS, non-high grade serous ovarian cancers; HGS, high grade serous ovarian cancers. Horizontal red bar denotes mean. ns not significant; * p<0.05; ** p<0.01; *** p<0.001, Mann-Whitney U test compared to HGS.


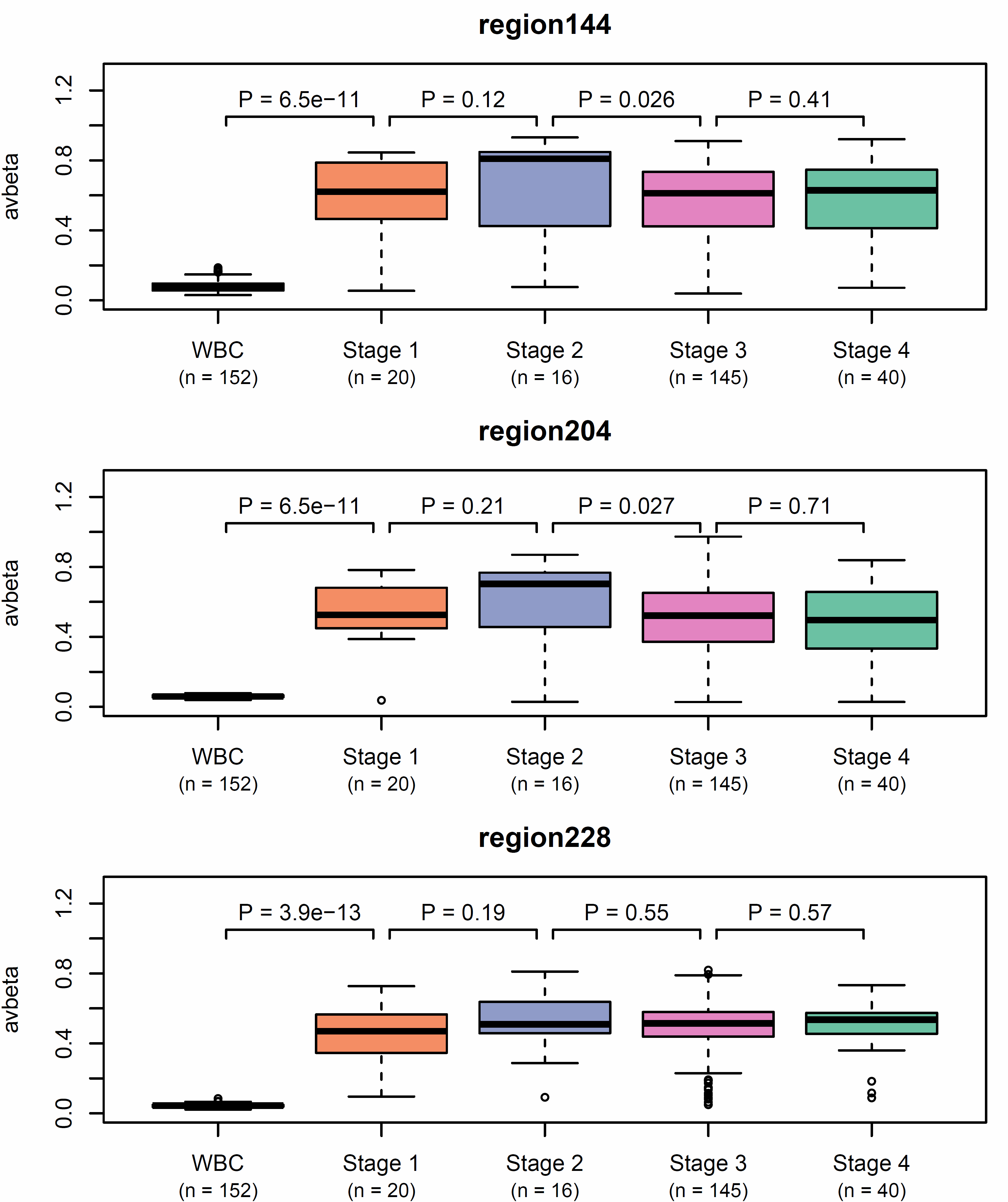


**Fig S6. Boxplots comparing the average beta values for 450k array probes within region #144 (average beta values cg21790626; cg27049766), #204 (average beta values cg15015892 and cg05021743) and #228 (cg22344703) between white blood cells (WBC) and different ovarian cancer stages (based on data from GSE72021).** P-values are from two tailed Wilcoxon rank sum test.

**Fig S7. Coverage (number of reads) for the three different regions analyzed in Serum Set 3 samples.** H, Healthy; BPM, benign pelvic mass; BOT, borderline tumor; NET, non-epithelial tumors, OCM, other cancerous malignancies; NHGS, non-high grade serous ovarian cancers; HGS, high grade serous ovarian cancers. Horizontal red bar denotes mean. ns not significant; * p<0.05; Mann-Whitney U test compared to HGS.

**

**

**Fig S8. CA125 levels measured in NACT Serum Set samples.** Samples taken before chemotherapy, after the first cycle of chemotherapy, and after the second cycle of chemotherapy. ns not significant; ** p<0.01; Mann-Whitney U test compared to before chemotherapy.





**Fig S9. Pattern frequencies for the top 3 reactions measured in NACT Serum Set samples**. Samples taken before chemotherapy, after the first cycle of chemotherapy, and after the second cycle of chemotherapy. * p<0.05; ** p<0.01; ** p<0.01; Mann-Whitney U test compared to before chemotherapy.

**Fig S10. Coverage (number of reads) for the top 3 reactions measured in NACT Serum Set samples.** Samples taken before chemotherapy, after the first cycle of chemotherapy, and after the second cycle of chemotherapy. ns not significant; Mann-Whitney U test compared to before chemotherapy.

**Fig S11. Average DNA amount extracted correlates with average UK temperature.** Boxplot of DNA amount extracted from UKCTOCS sample set (a different set which we analyzed to validate a breast cancer marker), collected at certain months of the year. Blue line represents average monthly UK temperatures (average UK data from 1981-2010 data set; metoffice.gov.uk)

**Fig S12. The fraction (%) of small fragment [50-250 base pairs (bp)] DNA in the serum DNA preparation for 171 UKCTOCS samples analyzed in the study.** The DNA fragment size and concentration were measured utilizing a Fragment Analyzer (AATI). For one sample the fragment analysis failed for technical reasons.

**(a)**


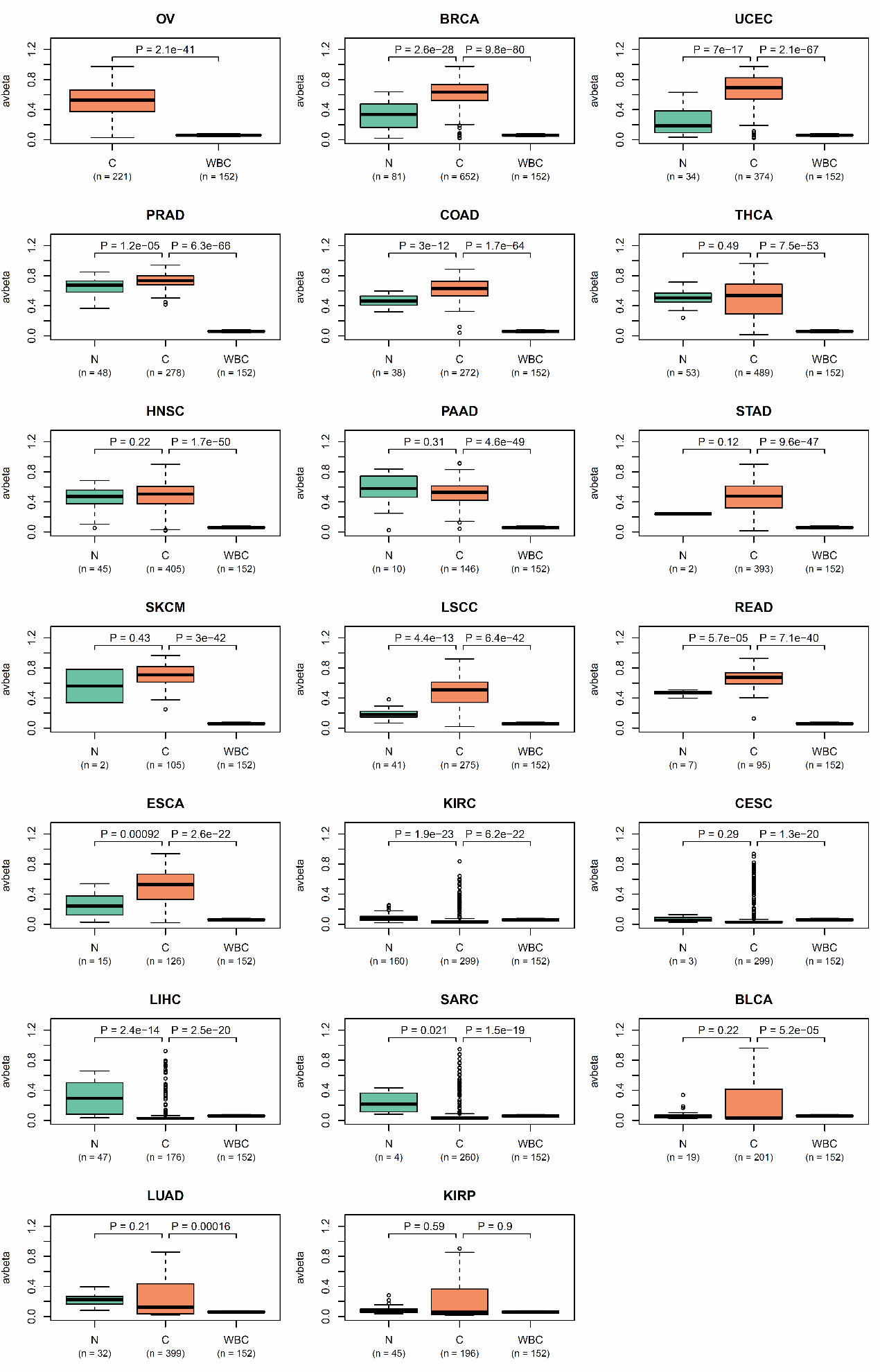


**
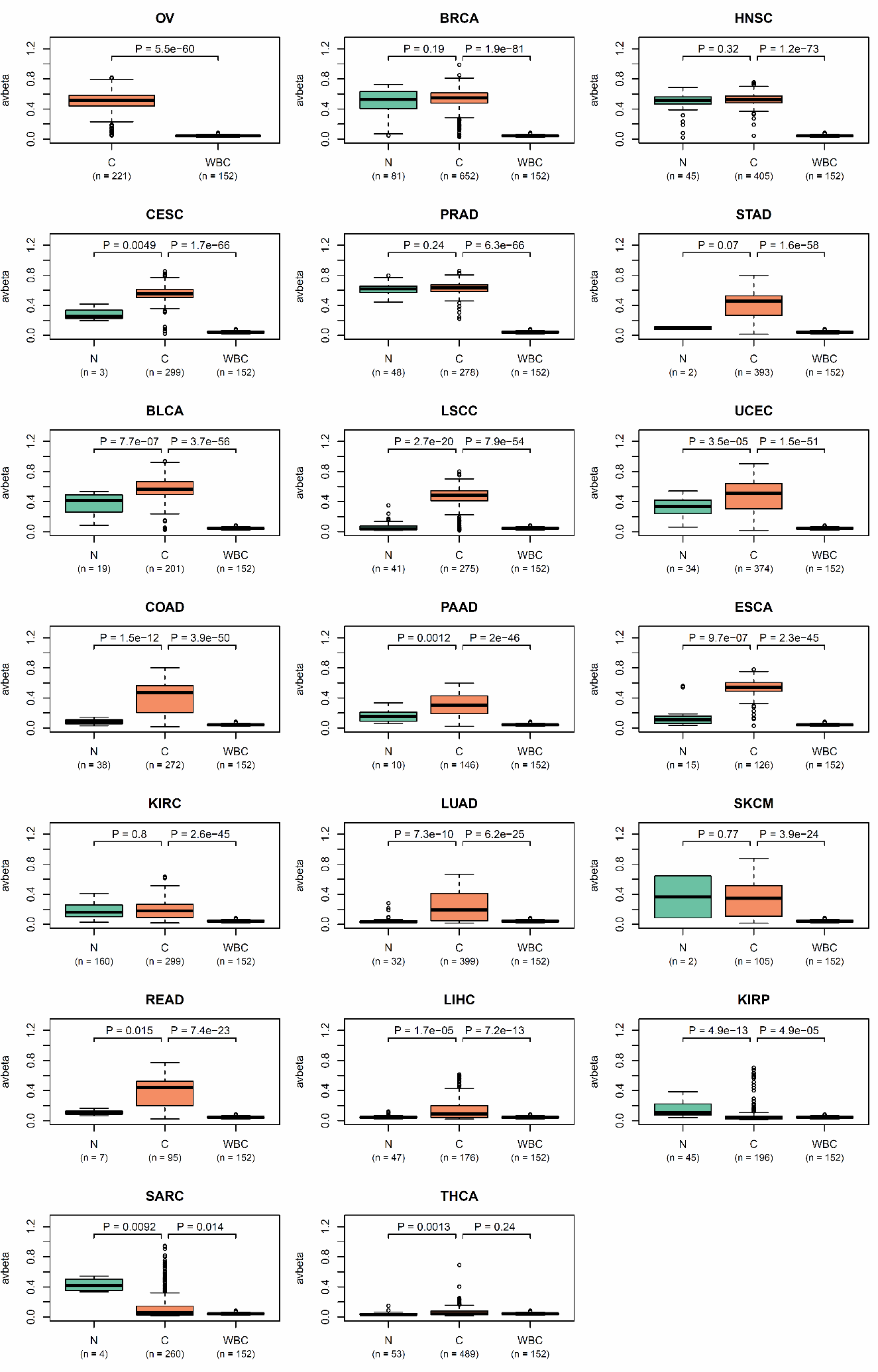
(b)**

**Fig S13. Boxplots comparing the average beta values for 450k array probes within region #204 (average beta values cg15015892 and cg05021743) (a) and #228 (cg22344703) (b) between each normal (N), cancer (C) group and white blood cell (WBC) data for ovarian cancer (GSE72021) and other 19 TCGA cancer types:** Bladder Urothelial Carcinoma (BLCA), Breast invasive carcinoma (BRCA), Cervical squamous cell carcinoma and endocervical adenocarcinoma (CESC), Colon adenocarcinoma (COAD), Esophageal carcinoma (ESCA), Head and Neck squamous cell carcinoma (HNSC), Kidney renal clear cell carcinoma (KIRC), Kidney renal papillary cell carcinoma (KIRP), Liver hepatocellular carcinoma (LIHC), Lung squamous cell carcinoma (LSCC), Lung adenocarcinoma (LUAD), Pancreatic adenocarcinoma (PAAD). Prostate adenocarcinoma (PRAD), Rectum adenocarcinoma (READ), Sarcoma (SARC), Skin Cutaneous Melanoma (SKCM), Stomach adenocarcinoma (STAD), Thyroid carcinoma (THCA), Uterine Corpus Endometrial Carcinoma (UCEC). P-values are from two tailed Wilcoxon rank sum test.
