# Supplementary material for: The potential of circulating tumor DNA methylation analysis for the early detection and management of ovarian cancer
Source: Genome Med. 2017 Dec 22;9:116. doi: 10.1186/s13073-017-0500-7 (PMC5740748; doi:10.1186/s13073-017-0500-7)
Supplement: Supplementary file 2 — Supplementary Material and Methods: Additional details of samples sets, methods, and analyses. (DOCX 75 kb) [file 13073_2017_500_MOESM2_ESM.docx]

**Supporting information for:**

**The potential of circulating tumor DNA methylation analysis for the early detection and management of ovarian cancer**

**Supplementary Material and Methods:** Additional details of samples sets, methods and analyses.

**DNA methylation analyses in tissue samples:**

DNA was isolated from tissue samples using the Qiagen DNeasy Blood and Tissue Kit (Qiagen Ltd, UK, 69506) and 600ng was bisulfite converted using the Zymo methylation Kits (Zymo Research Inc, USA, D5004/8).

**Illumina Infinium Human Methylation450 BeadChip Array data analysis**

Genome wide methylation analysis was performed using the Illumina Infinium Methylation 450K beadchip (Illumina Inc USA, WG-314-1003). The raw data processing and quality control was performed in R/Bioconductor (versions 2.15.0/2.11) [1] using minfi [2] and BMIQ [3] packages. Identification of differentially methylated regions (DMRs) was carried out using Genedata Expressionist® for Genomic Profiling as described below.

To correct for the individual probe to probe variation in the affinity/sensitivity to unmethylated vs. methylated DNA we used fully methylated (SssI-treated) and unmethylated (whole genome amplified; WGA) genomic DNA from WBC samples of different individuals. These technical controls were used for both filtering out probes that do not show sufficient specificity (i.e. cannot discriminate between methylated vs. unmethylated state) and to perform array-wide recalibration of the biological sample data to normalize for the probe to probe variation in background and dynamic range, respectively. The removal of the non-specific probes was achieved by doing a t-test with SssI vs WGA samples (using M-values) and removing the probe sets that have p-value <0.01 and effect size below <5 i.e. cannot discriminate between fully methylated and unmethylated DNA. The normalization was performed for each sample individually for each probe set with the formula M_true_= (M_measured_- M_WGA)_ / (M_SssI-_ M_WGA_); M_SssI_ and M_WGA_ values used were average (arithmetic mean) values of the respective sample groups. For the downstream analyses the individual sample SssI and WGA data were also normalized with the same formula. This leads to efficient removal of background noise from the probe to probe variation and increases the power to detect homogenously methylated or unmethylated genomic regions. T-test and normalization were performed in Genedata Expressionist® for Genomic Profiling software.

The control sample set was selected to identify DMRs that are cancer specific and would also be specific in a serum based clinical assay. Therefore, in addition to the ovarian (including Fallopian Tube and endometrium) control tissues we used a large panel of tissues that are likely to shed DNA into the serum [i.e. white blood cells (WBCs), lung, liver, rectum and colon], with WBCs being the most abundant source of normal germline DNA in serum samples. Two statistical approaches were used to identify DMRs from the 450K data: (1) a statistical test to identify single probes showing differential methylation between ovarian cancer and WBC samples, and (2) a sliding window ANOVA approach that scans the whole genome and identifies sets of neighboring probes (Ranges) showing correlated methylation differences between ovarian cancer and WBC samples. Only the DMRs showing no methylation in WBCs were considered for downstream analysis steps. The identified DMRs where ranked and scored based on the following criteria: (1) Differences in methylation levels between ovarian cancer and the control tissues (with WBC difference being emphasized). (2) Feasibility of designing a clinical assay (number of CpGs in the region to allow an assay to be designed with sufficient sensitivity/specificity). (3) For ranges only: Reliability of the DMRs (number of probes within the Range).

In the sliding window approach, the algorithm performs a pooling of all features in a given sliding window (120bp) before it calculates an ANOVA p-value between sample groups. The pooling increases statistical robustness and also results in smoother ANOVA p-values. The smoothed ANOVA p-values are then used to detect regions containing one or more p-values exceeding the given Maximum p-value threshold (1e-5). If a gap of more than 1000bp is detected between similar methylation differences, two different regions are reported. Note, that the algorithm also reports single probes showing significant methylation difference (if no neighboring similar methylation difference is present), but groups of probes with a similar profile do get lower p-values and are therefore preferentially reported. The sliding window approach was used for OC vs WBC samples to detect cancer DMRs (using normalized M-values) and arithmetic mean M-values of the probes per detected DMR (hereafter referred to as “Range”) were reported for all the relevant samples for downstream analysis. The Range discovery was performed in Genedata Expressionist® for Genomic Profiling v8.0 software. The Ranges varied in size between 1 and 45432bp, with average (arithmetic mean) size being 368bp. The Ranges showing methylation in WBC were removed by a t-test with WBC vs. WGA samples (p-value <1e-6 and/or directed effect >0.15; i.e. M(WBC) > M(WGA)+0.15). Next, a t-test for OC vs WBC was used and Ranges showing significant difference (p-value <1e-6) and difference (directed effect) of WBC upper quartile vs OC lower quartile >0.15 were selected as differentially methylated regions. For different control tissue samples the methylation values were calculated for the same OC vs WBC Ranges (arithmetic mean M-values of the probes per detected DMR).

For ranking of the DMRs, the effect sizes of methylation of cancer samples versus different relevant control tissues were calculated. In addition to “direct” control tissues (fimbrial/endometrial/benign ovarian tissues), large tissues with high turnover (liver, lung, rectum and colon) were also included (Table 1); data were download from the TCGA data portal (<https://tcga-data.nci.nih.gov/tcga/>) as level 3 data as detP filtered beta-values; data normalization was carried out as described above. The effect sizes were always calculated with cancer lower quartile vs control tissue upper quartile values (based on SssI/WGA normalized M-values).

Two different scoring methods were used for the effect sizes. In Method 1 the OC vs WBC effect size was assigned the weight 6x, and all the control tissue 1/4x. In Method 2 the OC vs WBC effect size was assigned the weight 6x, and all the control tissue 1x. Method 2 takes more into account with respect to the data from all the control tissues whereas Method 1 maximizes the effect of the difference between WBC and cancers samples. However, for both methods only DMRs pre-filtered for low methylation in WBCs were used (as described above). The final scores are the sum of the tissue scores and the feasibility and confidence scores (see next paragraph). If data were not available for a certain probe for a certain tissue (i.e. was filtered out due to high detection p-value), the score for the tissue was 0.

For further ranking of the DMRs feasibility (for designing a functional clinical assay) and confidence (for ranges) scores were calculated. The feasibility score is based on number of CpG dinucleotides within (or close by; +/- 60 bp) a probe/range. If the number of CpGs is <5, the score is -0.5, if the number of CpGs is between 5 and 9 the score is 0 and if the number of CpGs is >=10 the score is 0.5. The number of CpGs per range was calculated using EMBOSS cpgreport tool in Galaxy[4-6] using the range genomic coordinates as input. The confidence score for ranges is 0.5 if 2 or more probes are within the range, if only one probe the score is 0.

**Reduced Representation Bisulfite Sequencing (RRBS):**

*RRBS*

RRBS libraries were prepared by GATC Biotech using INVIEW RRBS-Seq according to proprietary SOPs. In brief, DNA was digested with the restriction endonuclease MspI that is specific for the CpG containing motif CCGG. The digested DNA is then adapter ligated, bisulfite modified and PCR-amplified. A size selection provides enhanced coverage for the CpG-rich regions including CpG islands, promoters and enhancer elements [7, 8]. The libraries were sequenced on Illumina’s HiSeq 2500 with 50 bp or 100 bp paired-end mode.

After sequencing raw data was trimmed using Trimmomatic (0.32)[9] to remove adapter sequences and low quality bases at the beginning and end of reads.

Subsequently, reads were trimmed with TrimGalore (0.3.3) (<http://www.bioinformatics.babraham.ac.uk/projects/trim_galore/>) to remove cytosines derived from library preparation which must not be included in the methylation analysis. Read pairs were mapped to the human genome (hg19) in Genedata Expressionist® for Genomic Profiling 8.0 applying Bisulfite Mapper based on BOWTIE v2.1.0 [10] with the settings --no-discordant --reorder -p 8 --end-to-end --no-mixed -D 50 -k 2 --fr --norc -X 400 -I 0 --phred33. Further analysis was carried out using Genedata Expressionist® for Genomic Profiling v9.1.

*Computation of methylation pattern frequencies*

In order to allow for the sensitive detection of methylation patterns with low abundance, the read data available for each sample type (e.g. breast cancer, ovarian cancer and white blood cells) were pooled across patients and sequencing runs. Candidate genomic regions for methylation pattern analysis were defined based on bundles of at least 10 paired-end reads covering at least 4 consecutive CpG sites which are located within a genomic range of, at most, 150 bp. As illustrated in the Figure below, our algorithm first determines sets of consecutive CpG sites of maximum size, from which multiple, potentially overlapping subsets are derived, which still meet the selection criteria. CpG sites located in the gap between the mate reads are ignored. For each derived set of CpG sites, the absolute and relative frequencies of all methylation patterns observed in the corresponding reads are determined. The methylation patterns are represented in terms of binary strings in which the methylation state of each CpG site is denoted by 1 if methylated or 0 if unmethylated. The algorithm for selecting candidate regions and calculating methylation pattern frequencies was implemented in our software platform Genedata Expressionist^®^ for Genomic Profiling.


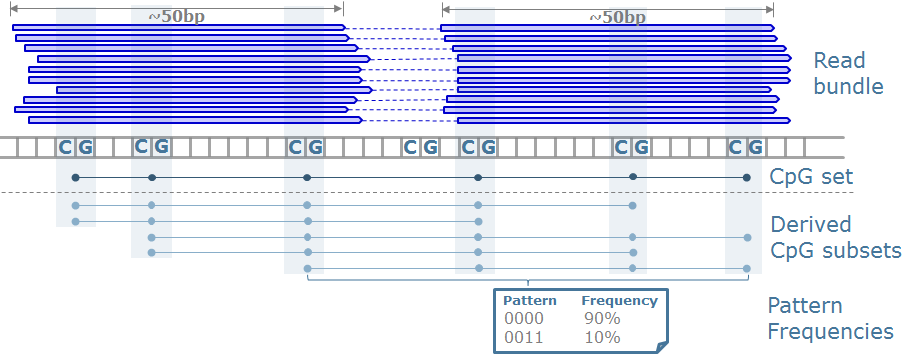


*Procedure for the selection of tumor-specific patterns*

In order to ensure that the pattern exclusively occurs in tumor samples, all patterns present in white blood cells were excluded. A score for assessing the relevance of each pattern was determined by integrating multiple subordinate scores which quantitatively capture desired properties of candidate biomarker patterns. First, for each pattern a Tumor Specificity Score $S_{P}=DL\cdot TP\cdot TE\cdot AF$ was calculated, which consists of the four components Dilution Factor *DL*, Tumor Prevalence *TP*, Tumor Enrichment Factor *TE* and Avoiding Factor *AF*. The formal definitions of the score components are given in the following:

$${DL}_{WBC}=\frac{\#total reads}{\#reads with pattern}*\frac{1}{{10}^{3}}$$

$${TP}_{tumor}=\frac{\#reads with pattern in tumor}{\#total reads in tumor}*10$$

$${TE}_{tumor}=\frac{\#observed reads with pattern in tumor}{\#expected reads with pattern in tumor}$$

$${AF}_{WBC}=\frac{\#expected reads with pattern in WBC}{\#observed reads with pattern in WBC}$$

The Dilution Factor *DL* and Tumor Prevalence *TP* favor patterns which are supported by a high proportion of reads in tumor and low proportion of reads in WBC, respectively. A pattern observed in 1 out of 10 reads in tumor and in 1 out of 1000 reads in WBC scores 1 for both factors. The Tumor Enrichment Factor *TE* and Avoiding Factor *AF* were included to assess the overrepresentation of the pattern in tumor samples and its underrepresentation in WBC samples, respectively, relative to an expected number of pattern reads which is based on the observed overall methylation level in those tissues. In order to estimate the number of expected reads supporting the pattern, the methylation frequencies are calculated for each CpG site individually. Next, the number of expected reads with a specific pattern is calculated as the product of the relative frequencies of the tumor specific methylation states observed for each CpG site in the pattern times the number of reads stretching across the pattern. A TE >1 indicates that a pattern is more frequent in tumor than expected when randomly distributing the observed methylation levels across reads. Besides favoring tumor specificity our scoring procedure was also designed to make patterns with high variance of the highest priority (i.e., patterns for which a high number of transitions in the methylation state is observed between consecutive CpG sites). Such patterns may be a product of the epigenetic reprogramming of tumor cells and in order to account for the potentially increased biological relevance of these patterns another score component was introduced. The normalized variance $V_{P}$ of a pattern *p* is defined as the pattern variance divided by the maximum variance, i.e., the pattern length minus 1. The scores for the tumor specificity $S_{P}$ and pattern variance $V_{P}$ were combined in the tumor-specific variance score${SV}_{P}={V_{P}\cdot\log(S}_{P})$. In order to facilitate the ranking of each candidate genomic region *r* based on the relevance of patterns *p_1_,…, p_N_* observed in the region the aggregation score ${AS}_{r}$ was calculated based on the following formula:

$${AS}_{r}=\sum_{i=1}^{n} \frac{1}{i}{SV}_{Pi}$$

The aggregation score ${AS}_{r}$ corresponds to a weighted sum of the tumor-specific variance scores of the observed patterns. The weighting was included since an ordinary sum would introduce a bias towards regions, in which a high number of patterns have been observed due to a high read coverage and/or high CpG site density. All of the presented statistics for assessing the relevance of methylation patterns and genomic regions were implemented in Genedata Expressionist® for Genomic Profiling and R, respectively.

**DNA methylation analyses in serum samples:**

**Serum separation:**

For Serum Sets 1-3 and the NACT Serum Set, women attending the hospitals in London and Prague have been invited, consented and 20-40 mL blood has been obtained (VACUETTE® Z Serum Sep Clot Activator tubes, Cat 455071, Greiner Bio One International GmbH), centrifuged at 3,000rpm for 10 minutes and serum collected and stored at -80°C. We have applied non-stringent measures (i.e. allowed for up to 12 hours between blood draw and centrifugation) purposely in order to mimic the situation of UKCTOCS samples which have been sent from the recruiting centre to UCL within 24-48 hours before centrifugation.

**Serum DNA isolation and bisulfite modification:**

DNA was isolated at GATC Biotech (Konstanz, Germany). Serum DNA was quantified using the Fragment Analyzer and the High Sensitivity Large Fragment Analysis Kit (AATI, USA). DNA was bisulfite converted at GATC Biotech.

**Targeted ultra-high coverage bisulfite sequencing:**

Targeted bisulfite sequencing was performed at GATC Biotech. To this end, a two-step PCR approach was used similar to the recently published BisPCR2 [11]. Bisulfite modification was performed with 1mL serum equivalent. For each batch of samples, positive and non-template controls were processed in parallel. Bisulfite converted DNA was used to test up to three different markers using automated workflows. After bisulfite modification the target regions were amplified using primers carrying the target specific sequence and a linker sequence. Amplicons were purified and quantified. All amplicons of the same sample were pooled equimolarly. In a second PCR, primers specific to the linker region were used to add sequences necessary for the sequencing and multiplexing of samples. Libraries were purified and quality controlled. Sequencing was performed on Illumina’s MiSeq or HiSeq 2500 with 75 bp or 125 bp paired-end mode.

**Assessment of pattern frequency in serum DNA:**

After sequencing, raw data were trimmed using Trimmomatic (0.32) to remove adapter sequences and low quality bases at the beginning and end of reads. Further analysis was carried out using Genedata Expressionist® for Genomic Profiling 9.1. Read pairs were mapped to the human genome (hg19) applying Bisulfite Mapper based on BOWTIE v2.2.5 [10] with the settings --no-discordant -p 8 --norc --reorder -D 50 --fr --end-to-end -X 500 -I 0 --phred33 -k 2 --no-mixed. Coverage was calculated per sample and target region using Numeric Data Feature Quantification activity by calculating the arithmetic mean of the coverage in each region. As part of the data quality control, efficiency of the bisulfite conversion was estimated in each sample by quantifying the methylation levels of CpHpG and CpHpH sites (where H is Any Nucleotide Except G), with minimum coverage of 10, within the target regions. The median bisulfite conversion efficiency was 99.4%, with efficiency for no sample being lower than 97.7%. Methylation pattern frequencies in serum samples for target regions were determined as described above. Relative pattern frequencies were calculated by dividing the number of reads containing the pattern by the total number of reads covering the pattern region.

**SUPPLEMENTARY REFERENCES**

1. Gentleman RC, Carey VJ, Bates DM, Bolstad B, Dettling M, Dudoit S, Ellis B, Gautier L, Ge Y, Gentry J *et al*: **Bioconductor: open software development for computational biology and bioinformatics**. *Genome Biol* 2004, **5**(10):R80.

2. Aryee MJ, Jaffe AE, Corrada-Bravo H, Ladd-Acosta C, Feinberg AP, Hansen KD, Irizarry RA: **Minfi: a flexible and comprehensive Bioconductor package for the analysis of Infinium DNA methylation microarrays**. *Bioinformatics* 2014, **30**(10):1363-1369.

3. Teschendorff AE, Marabita F, Lechner M, Bartlett T, Tegner J, Gomez-Cabrero D, Beck S: **A beta-mixture quantile normalization method for correcting probe design bias in Illumina Infinium 450 k DNA methylation data**. *Bioinformatics* 2013, **29**(2):189-196.

4. Dedeurwaerder S, Defrance M, Calonne E, Denis H, Sotiriou C, Fuks F: **Evaluation of the Infinium Methylation 450K technology**. *Epigenomics* 2011, **3**(6):771-784.

5. Goecks J, Nekrutenko A, Taylor J: **Galaxy: a comprehensive approach for supporting accessible, reproducible, and transparent computational research in the life sciences**. *Genome Biol* 2010, **11**(8):R86.

6. Giardine B, Riemer C, Hardison RC, Burhans R, Elnitski L, Shah P, Zhang Y, Blankenberg D, Albert I, Taylor J *et al*: **Galaxy: a platform for interactive large-scale genome analysis**. *Genome Res* 2005, **15**(10):1451-1455.

7. Gu H, Smith ZD, Bock C, Boyle P, Gnirke A, Meissner A: **Preparation of reduced representation bisulfite sequencing libraries for genome-scale DNA methylation profiling**. *Nat Protoc* 2011, **6**(4):468-481.

8. Lee YK, Jin S, Duan S, Lim YC, Ng DP, Lin XM, Yeo GS, Ding C: **Improved reduced representation bisulfite sequencing for epigenomic profiling of clinical samples**. *Biol Proced Online* 2014, **16**(1):1.

9. Bolger AM, Lohse M, Usadel B: **Trimmomatic: a flexible trimmer for Illumina sequence data**. *Bioinformatics* 2014, **30**(15):2114-2120.

10. Chen PY, Cokus SJ, Pellegrini M: **BS Seeker: precise mapping for bisulfite sequencing**. *BMC Bioinformatics* 2010, **11**:203.

11. Bernstein DL, Kameswaran V, Le Lay JE, Sheaffer KL, Kaestner KH: **The BisPCR(2) method for targeted bisulfite sequencing**. *Epigenetics Chromatin* 2015, **8**:27.
