# Supplementary material for: The potential of circulating tumor DNA methylation analysis for the early detection and management of ovarian cancer
Source: Genome Med. 2017 Dec 22;9:116. doi: 10.1186/s13073-017-0500-7 (PMC5740748; doi:10.1186/s13073-017-0500-7)
Supplement: Supplementary file 3 — Coordinates and primers to amplify the identified target region. Table S2. Performance of the serum DNA methylation marker panel in a population-based cohort to diagnose OC early. Table S3. Direct comparison of the performance of the CA125 and the three-marker DNAme panel to predict OC development in the UKCTOCS cohort. (DOCX 29 kb) [file 13073_2017_500_MOESM3_ESM.docx]

**Supporting information for:**

**The potential of circulating tumor DNA methylation analysis for the early detection and management of ovarian cancer**

**Supplementary Tables**

| **#** | **Data basis** | **Coordinates** | **Primer sequence 1** | **Primer sequence 2** | **Amplicon size** |
| --- | --- | --- | --- | --- | --- |
| 123 | RRBS | chr16:1271152-1271271 | GYGAAGTAGGAGTAGTTGTYGGGTTTTA | TCTCTCCTAACRAAAAACTAACCCAATTACC | 120 bp |
| 129 | RRBS | chr11:69054638-69054757 | AATTTTGTTGAGTGAGTTTATAAATAGGGTATAA | TCACTTAACACCRTTTTTACTAACC | 120 bp |
| 137 | RRBS | chr12:132896275-132896404 | ATTTYGTTATATATATAGTTGTATTYGGTATAATA | CRAAAAACTAAACACCCAAACC | 130 bp |
| 141 | RRBS | chr5:178004395-178004530 | TATTYGGAGGTTTAGGGGTGAGGATTT | CAACRAATTCCCCRAATCCTATCCT | 136 bp |
| 144 | RRBS | chr19:58220413-58220552 | TTTAGGTTTGAYGTGGGTTTTTTAG | TCTAAAATATATTCACCRAATCAAAAATAACAAAA | 140 bp |
| 148 | RRBS | chr2:72359599-72359718 | GAGGTAATGGAAGYGGTTATTTTTG | ATATATAACATCCRAAACACCCATAACACAA | 120 bp |
| 150 | RRBS | chr7:156735029-156735165 | GYGGGTATTTGTAGTTTTAGTTATT | TTTTTAAACRAAATCTCRCTCTAT | 137 bp |
| 154 | RRBS | chr17:70112132-70112268 | TTTTYGGAGTTYGGAGTTTAGGTTAGTGGTA | AAAACRCATCTCCCTAACTCCTACAAACTA | 137 bp |
| 158 | RRBS | chr16:74441696-74441831 | GATTTTGTTTTAAAAAAGAAAAAAATAGGGT | AAATTTCACCRTATTACCCAAAATAATAT | 136 bp |
| 164 | RRBS | chr4:174427917-174428054 | GATTYGYGAGGTTTTTTAGTAGTTTATTT | AAACRACRATTCCCAACATCTAAACT | 138 bp |
| 176 | RRBS | chr6:119107203-119107340 | TAATTTATTTTTTTATTAAATTGTATGAAGAAGGT | AATAAAAACRAAATTTCACCRTAATCT | 138 bp |
| 178 | RRBS | chr19:13215409-13215550 | GGTAGGAGYGTTTTATTATGYGTAAGTT | CCAAAACCRAAACCAACRATTCC | 142 bp |
| 180 | RRBS | chr3:192125846-192125980 | TTGTAGAAGYGTATTTTGTTGAATATTTYGAGGA | ACAAAAACRACAAACRAAAAAATCCAACAA | 135 bp |
| 186 | RRBS | chr22:21483239-21483384 | YGTGTTAGTTAGGATGGTTTYGATTTTTTGATTT | TCCCAAAATACCACAACCCAAACC | 146 bp |
| 188 | RRBS | chr19:18497131-18497271 | AGAGTTGTATTTYGAAGATTTTAGATTT | AACRTAAATATCCRAACTACAAAAAC | 141 bp |
| 190 | RRBS | chr9:79629064-79629172 | GTTAGAYGAGAGTTTGGGGTTAATGT | AAATCCAACCAACAACRAAAAACCAAA | 109 bp |
| 192 | RRBS | chr12:75601294-75601437 | YGGTAGGTTATTTAGTAGTAGGGTTTTA | CRTCTTCRCCTATATACTCAATTACTAC | 144 bp |
| 200 | RRBS | chr9:138999180-138999294 | AATTAGTTTAGTAATYGGYGATTTTAAG | CCACTAACAAATAAATCCTATATTCCTAC | 115 bp |
| 202 | RRBS | chr1:2987508-2987655 | GTGYGAATAAGATYGGGYGTTT | CRATAATCCCRACTCTAAACAAACC | 148 bp |
| 204 | RRBS | chr1:151810784-151810937 | GATATTYGGTGGAGAGTYGTAGTTGTT | ACCRATACCAACCCRCACTC | 154 bp |
| 208 | 450K | chr8:55467518-55467638 | ATATTAATTTTGTTYGGGTAYGGTGGTTT | CRAAATTTCACCATATTCRCCAAAATAATCT | 121 bp |
| 210 | 450K | chr12:123713499-123713590 | GGAGTTGTAAAAAATAAAGGAATATGTG | TACATACAAATTACTATAAAACCATTTCCTATAC | 92 bp |
| 213 | 450K | chr2:106776938-106777040 | TYGTTYGGGAATGGGAATATAGTTATATATGG | CCRAATACACACTCCACAATCC | 103 bp |
| 214 | 450K | chr3:141516260-141516353 | TTGTTTAAAGGYGTAGAGGAGTAGTTGG | TCAACTATTTCCTAAAACTCTCTCCTTCCTTC | 94 bp |
| 219 | 450K | chr16:30484157-30484257 | GGATGAAGGATTTTTGTATTATTGTGATGGTTATG | AAACACATATCCCTTCCCCTACCTC | 101 bp |
| 222 | 450K | chr3:111809437-111809506 | TAGGTTATAGGAAGAGGTATTTTTTATAGATG | ACTTCCTAAAAAAACTCAACTTAAAATTTTAC | 70 bp |
| 223 | 450K | chr10:120489250-120489333 | AAGAGAGAGTGGTTGATAATTAGTAG | ACAACTAATATACAAAAAAATTATATTACAAACAC | 84 bp |
| 224 | 450K | chr11:1874037-1874133 | GGTTTTTTTTTTYGAGTTATGAAGAGTTG | AAACRTTAAACAAATTACAAAAACCTAAAATAAC | 97 bp |
| 225 | 450K | chr7:142422193-142422278 | GAAGTTTGATATTTTTGGTTTTAAATATTGTTTG | CCACAACTTACTATACATAAAAAATATCTATCC | 86 bp |
| 226 | 450K | chr1:3086452-3086542 | GGGGGGATTGTYGTTAATTTATTGTTTAATG | CCACRAAATATACACAATCCACATACATCAC | 91 bp |
| 228 | 450K | chr2:219736276-219736386 | GTTTTATGGGYGAGTTGTTGTAGTG | AAAAACCCRCRAAACCAAAAAATC | 111 bp |

**Table S1: Coordinates and primers to amplify the identified target region**.

|  | **Three DNAme-Marker Panel (Original Threshold)** | | | | | |
| --- | --- | --- | --- | --- | --- | --- |
|  | All Samples | | | CA125 negative samples | | |
|  | 0-2 years | 0-1 years | 1-2 years | 0-2 years | 0-1 years | 1-2 years |
| Specificity | 125/129 | 125/129 | 125/129 | 4/125 | 4/125 | 4/125 |
| Sensitivity | 10/43 | 6/19 | 4/24 | 4/26 | 2/9 | 2/17 |
|  |  |  |  |  |  |  |
|  | **Three DNAme-Marker Panel (3-fold lower Threshold),**  **Low DNA amount samples** | | | | | |
|  | All Samples | | | CA125 negative samples | | |
|  | 0-2 years | 0-1 years | 1-2 years | 0-2 years | 0-1 years | 1-2 years |
| Specificity | 59/67 | 59/67 | 59/67 | 56/64 | 56/64 | 56/64 |
| Sensitivity | 11/19 | 5/9 | 6/10 | 7/11 | 4/6 | 3/5 |
|  |  |  |  |  |  |  |
|  | **Three DNAme-Marker Panel (3-fold lower Threshold),**  **High DNA amount samples** | | | | | |
|  | All Samples | | | CA125 negative samples | | |
|  | 0-2 years | 0-1 years | 1-2 years | 0-2 years | 0-1 years | 1-2 years |
| Specificity | 56/62 | 56/62 | 56/62 | 55/61 | 55/61 | 55/61 |
| Sensitivity | 3/24 | 3/10 | 0/14 | 0/15 | 0/3 | 0/12 |

**Table S2: Performance of the serum DNA methylation marker panel in a population based cohort to diagnose ovarian cancer early.**

The pattern frequency cut-off for #141, #204 and #228 is 0.00027, 0.00001 and 0.0000033, respectively. A test was called positive if at least one of the three markers was above this pre-specified threshold. Specificity and Sensitivity are shown for all samples as well as for samples with low (below the median) and high DNA amount depending on time between sample donation and diagnosis of disease as well as CA125 status (< 35IU/mL).

| **Three DNAme-Marker Panel (3-fold lower Threshold), Low DNA amount samples** | | | |
| --- | --- | --- | --- |
|  | 0-2 years | 0-1 years | 1-2 years |
| Specificity | 88.1% (59/67) | 88.1% (59/67) | 88.1% (59/67) |
| Sensitivity | 57.9% (11/19) | 55.6% (5/9) | 60.0% (6/10) |
|  |  |  |  |
| **CA125 (cut off 35 IU/ml), Low DNA amount samples** | | | |
|  | 0-2 years | 0-1 years | 1-2 years |
| Specificity | 95.5% (64/67) | 95.5% (64/67) | 95.5% (64/67) |
| Sensitivity | 42.1 (8/19) | 33.3% (3/9) | 50% (5/10) |

**Table S3: Direct comparison of the performance of the CA125 and the three-marker DNA methylation panel to predict ovarian cancer development in the UKCTOCS cohort.**
